# Targeted Multifunctional Fluorine‐Rich Copolymer Coating Design for Ambient‐Stable Prelithiated SiOC Anodes

**DOI:** 10.1002/advs.76562

**Published:** 2026-08-03

**Authors:** Rong Chen, Yixuan Fan, Congcong Zhang, Yinan Liu, Yun Zheng, Ruifeng Zheng, Yingying Shen, Pingshan Jia, Luojiang Zhang, Yongbing Tang, Huaiyu Shao

**Affiliations:** ^1^ Institute of Applied Physics and Materials Engineering University of Macau Avenida da Universidade Macao SAR China; ^2^ Advanced Energy Storage Technology Research Center Shenzhen Institutes of Advanced Technology Chinese Academy of Sciences Shenzhen China; ^3^ University of Chinese Academy of Sciences Beijing China

**Keywords:** electrolyte compatibility, fluorine‐rich copolymer, hydrophobicity, multifunctional coating, prelithiated electrode protection

## Abstract

Prelithiation is a pivotal strategy for enhancing the initial coulombic efficiency (ICE) and energy density of lithium‐ion batteries, yet its practical application is impeded by the pronounciked sensitivity of prelithiated electrodes to ambient moisture and oxygen during storage. Herein, we rationally devise a targeted design for a fluorine‐rich acrylate copolymer—poly (tridecafluorooctyl methacrylate‐co‐methyl methacrylate) (PFMMA)—and introduce it as a multifunctional protective coating, with Li_13_Si_4_‐prelithiated SiOC electrodes (preSiOC) employed as a proof of concept. Fluorinated side chains impart strong hydrophobicity, while methyl methacrylate units retain electrolyte affinity; the two moieties act synergistically to stabilize electrodes in air and preserve unimpeded interfacial ion/charge transport during redox reactions. Consequently, the preSiOC/PFMMA electrode with a 540 nm‐thick PFMMA coating retains 97.4% capacity and 95.3% ICE after 48 h air exposure at 50% relative humidity (RH), alongside robust cycling stability (677.5 mAh·g^−1^ after 100 cycles). These results outperform both unprotected preSiOC and other reported conventionally protected prelithiated electrodes. Furthermore, the electrode shows exceptional environmental adaptability, maintaining functionality under extreme scenarios (10% RH for 100 days or 90% RH for 3 days). This study establishes a rational copolymer design paradigm for fabricating durable, electrolyte‐compatible interfaces, thereby accelerating the development of ambient‐stable prelithiated electrodes.

## Graphical abstract

A fluorine‐rich acrylate copolymer, poly (tridecafluorooctyl methacrylate‐co‐methyl methacrylate) (PFMMA), is introduced as a multifunctional protective coating to stabilize the prelithiated SiOC electrodes in humid conditions and preserve unimpeded interfacial ion/charge transport during redox reactions.

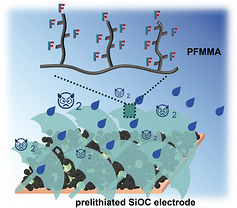

## 1 Introduction

Rechargeable lithium‐ion batteries (LIBs) have undergone revolutionary advancements in consumer electronics and emerged as critical components for portable electronic devices, electric vehicles, and grid‐scale energy storage systems [1, 2, 3, 4]. While graphite anodes offer a high ICE (>90%), their limited theoretical capacity (372 mAh·g^−1^) has driven growing interest in high‐capacity alternatives like silicon anodes (3579 mAh·g^−1^) [5, 6, 7, 8]. However, Si‐based anodes suffer from severe ICE losses (70%–85%) due to Li consumption during solid electrolyte interphase (SEI) formation and ∼300% volume expansion [9, 10]. Prelithiation (utilization of prelithiation additives, chemical/electrochemical/direct contact prelithiation, etc.) has emerged as a promising strategy to mitigate lithium inventory loss during initial SEI formation in LIBs, thereby enhancing the ICE and overall energy density [11, 12, 13, 14].

Despite achieving partial progress, prelithiation technologies continue to face persistent safety challenges spanning material handling, manufacturing protocols, and long‐term storage stability [15, 16, 17, 18]. Particularly problematic is the rapid electrochemical and morphological degradation of prelithiated electrodes upon exposure to trace oxygen or moisture, which triggers parasitic reactions including lithium oxidation and hydroxide formation [19, 20, 21]. Current mitigation strategies typically require maintaining relative humidity below 1% in dry‐room facilities across the entire production workflow, which substantially increases operating costs due to stringent dehumidification demands and can further constrain manufacturing throughput [22, 23]. These constraints underscore the imperative for developing air‐stable prelithiation architectures compatible with conventional battery fabrication infrastructures. At the materials level, for example, a Li_x_Si/Li_2_O core‐shell architecture was elaborately designed to enhance dry‐air stability of Li_x_Si alloy prelithiation additive particles [24]. Subsequent advancements addressed humid environments through artificial SEI formation via fluorodecane and Li_x_Si reactions, achieving 60% capacity retention after 6 h exposure at 40% relative humidity (RH) [25]. Despite progress, the large population of micro‐/nanoscale particles still hinders comprehensive and durable protection, thereby increasing the risk of environmental degradation and electrochemical performance decay. Surface passivation at the electrode level offers a scalable manufacturing route. Conformal encapsulation using hydrophobic polymers (e.g., poly(vinylidene–co–hexafluoropropylene), ethylene–vinyl acetate copolymer, etc.) has been revealed to offer partial moisture protection [26, 27]. However, these strategies mainly rely on relatively inert polymer matrices to isolate the prelithiation reagent from moisture and air. In contrast, the employment of electrolyte‐soluble coating layers can be feasible to mitigate this issue, but their insufficient structural density and hydrophobicity limit long‐term resistance to oxidative degradation and moisture ingress [28, 29]. Therefore, the present work focuses on the molecular design of the protective polymer itself, aiming to construct a fluorine‐rich acrylate layer that not only provides hydrophobic protection but also maintains interfacial compatibility with the battery electrolyte.

In this work, a fluorine‐rich acrylate copolymer, poly (tridecafluorooctyl methacrylate–co–methyl methacrylate) (PFMMA), is rationally designed as a multifunctional protective layer, using the Li_13_Si_4_‐prelithiated SiOC (preSiOC) anode as a proof of concept. This design was validated by both electrochemical and environmental‐stability tests. The preSiOC anode coated with a thin PFMMA layer (∼540 nm), denoted as preSiOC/PFMMA, delivers a specific capacity of 1457.2 mAh·g^−1^ and an ICE of 100.5%. Crucially, preSiOC/PFMMA maintains 97.4% capacity retention with an ICE of 95.3% after 48 h of air exposure at 50% RH. Remarkably, the anode retains 94.5% capacity after 100 days at 10% RH and 88.7% capacity after 3 days at 90% RH, demonstrating its superior interfacial protection capability over conventional strategies [24, 25, 26, 27, 28, 29, 30, 31, 32, 33, 34, 35].

## 2 Results and Discussion

### 2.1 Design and Synthesis of PFMMA

Fluorinated polymers are known for their high thermal and chemical stability, low surface energy, and strong hydrophobicity, making them ideal for protective coatings. In this work, 3,3,4,4,5,5,6,6,7,7,8,8,8–tridecafluorooctyl methacrylate (TFOA) is employed owing to its perfluoroalkyl moieties that confer pronounced hydrophobicity, a critical attribute for moisture barrier applications. However, poly(TFOA) homopolymer exhibits inherent limitations, including inadequate film‐forming properties and limited solubility in polar solvents such as ethylene carbonate, diethyl carbonate, and so on [36]. To overcome these limitations, methyl methacrylate (MMA) was introduced as a comonomer because of its favorable electrolyte compatibility and compatibility with scalable electrode manufacturing. PMMA‐based materials have been widely used in polymer and gel polymer electrolytes, reflecting their good adaptability to lithium electrochemical environments [37, 38]. It should be noted, however, that such compatibility does not necessarily imply complete insolubility in liquid electrolytes, as the dissolution behavior depends on the polymer structure, molecular weight, degree of crosslinking, and electrolyte composition. In general, high‐molecular‐weight MMA‐based polymers are more suitable for gel electrolyte frameworks, whereas reducing the molecular weight and polymer content can promote the dissolution behavior described in this work. On this basis, the resultant copolymer PFMMA is expected to achieve a tailored equilibrium between hydrophobicity, mechanical robustness, and interfacial compatibility, thereby establishing an essential critical protective interface for prelithiated electrodes.

**FIGURE 1:**
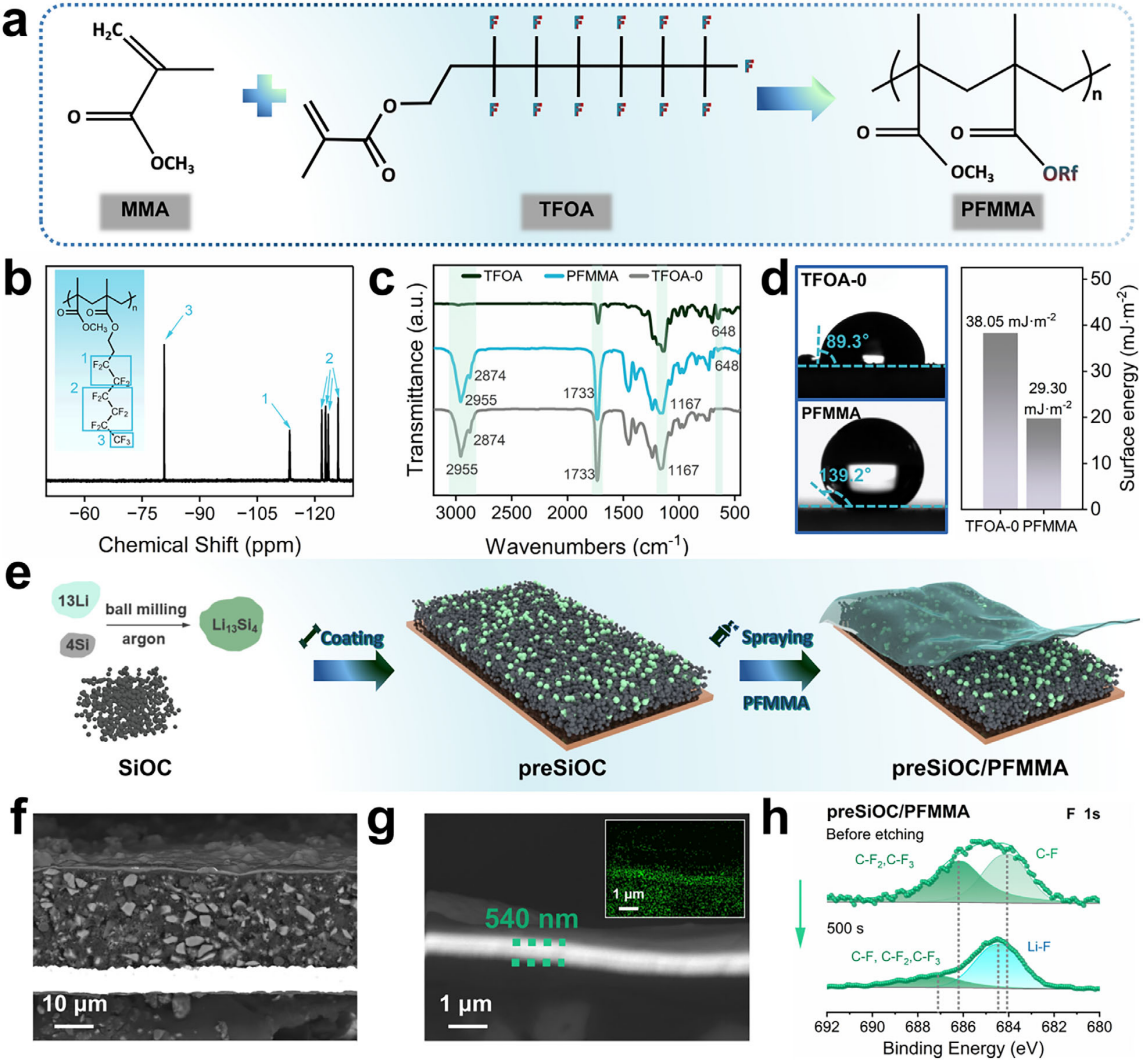
Structural design and characterization of the fluorine‐rich hydrophobic acrylate protective layer. (a) Schematic synthetic route of PFMMA. (b) ^19^F NMR spectrum of PFMMA. (c) FTIR spectra of TFOA, PFMMA, and the non‐fluorinated reference (TFOA‐0). (d) Static water contact angles and surface energy analysis of TFOA‐0 and PFMMA films. (e) Conceptual illustration of the prelithiation protection strategy imparted by the PFMMA coating. (f,g) Cross‐sectional SEM images of the preSiOC/PFMMA electrode. (insert: corresponding EDX mappings of F element) (h) XPS depth profile of the preSiOC/PFMMA electrode.

As shown in Figure 1a and Figure S1, PFMMA was first synthesized via free‐radical copolymerization of TFOA and MMA monomers. The polymerizable vinyl functionality in TFOA enables efficient copolymerization with MMA, permitting precise modulation of copolymer composition and functionality. The ^19^F nuclear magnetic resonance (NMR) spectrum of PFMMA (Figure 1b) confirms the successful incorporation of TFOA into the copolymer. The signal at −114.0 ppm corresponds to the characteristic resonance of fluorine atoms in –CH_2_–CF_2_– moieties, while the peak at −80.8 ppm is attributed to fluorine atoms in the terminal –CF_3_ group, and the broad signal ranging from −125.5 to −121.3 ppm is associated with fluorine atoms in internal –CF_2_(CF_2_)_2_CF_2_– segments [39]. Fourier transform infrared (FTIR) spectroscopy analysis further verifies PFMMA synthesis by identifying the characteristic vibrational modes associated with its functional groups. As depicted in Figure 1c, the absorption bands at 2955 and 2874 cm^−1^ correspond to the stretching vibrations of methyl (–CH_3_) and methylene (–CH_2_–) groups, respectively [40]. The strong peak at 1733 cm^−1^ is assigned to C═O stretching, while the 1167 cm^−1^ band arises from C─O─C stretching. The disappearance of the peak at 890 cm^−1^ (vinyl out‐of‐plane bending vibration) indicates the complete consumption of monomers. Comparative analysis with the non‐fluorinated control sample (TFOA‐0, synthesized only using MMA monomer under identical conditions) reveals a distinctive C‐F stretching vibration at 648 cm^−1^ in PFMMA, verifying covalent incorporation of TFOA [41]. Thermogravimetric analysis (TGA, Figure S2) demonstrates significantly enhanced thermal stability of PFMMA relative to TFOA‐0.

The as‐prepared PFMMA film is highly transparent with good film‐forming characteristics (Figure S3). Morphological analysis via scanning electron microscopy (SEM, Figure S4) reveals a microscopically smooth and homogeneous surface topography, with energy‐dispersive x‐ray spectroscopy (EDS) mapping confirming the uniform spatial distribution of fluorine element throughout the copolymer matrix. In addition, Figure 1d demonstrates that the incorporation of perfluorinated chain segments elevates the water contact angle from 89.3° to 139.2°, accompanied by a reduction in surface energy from 38.05 to 29.30 mJ·m^−2^ (Figure S5, Tables S1 and S2, calculation formulas refer to Supporting Information). The transformation can originate from synergistic mechanisms: (i) thermodynamic surface segregation of low‐energy perfluorinated groups establishing a protective barrier against polar interactions; and (ii) steric shielding of backbone functionalities through fluorine‐rich domain assembly.

### 2.2 Synthesis and Characterization of PFMMA‐Coated Prelithiated Anodes

PFMMA was successfully synthesized and applied as a protective coating on the electrode surface to improve environmental stability. The coating procedure of PFMMA layer on Li_13_Si_4_‐prelithiated SiOC (preSiOC) anode is schematically depicted in Figure 1e. The prelithiation additive Li_13_Si_4_ was synthesized via high‐energy ball milling of stoichiometric lithium/silicon mixtures. The main diffraction peaks in XRD (x‐ray Diffraction) profile (Figure S6) match well with the standard pattern of Li_13_Si_4_ (PDF Card No. 29–0830, ICDD), although some impurity peaks associated with Li_2_O and LiOH due to exposure in air during transfer can be observed. In the Li_13_Si_4_||Li half‐cell configuration, the Li_13_Si_4_ exhibits a high lithium extraction capacity of 1134 mAh·g^−1^ (Figure S7). Besides, the amount of Li_13_Si_4_ can be optimized to be 20 wt.% in the preSiOC anode with an active material loading of 50 wt.% based on the ICE results (Figure S8).

The as‐prepared PFMMA layer was deposited onto preSiOC via spray coating of copolymer/1,3–dioxolane (DOL) solution (Figure S9), yielding the PFMMA‐coated prelithiated electrodes (preSiOC/PFMMA) after the DOL was evaporated. The PFMMA mass loading can be precisely modulated from 0.05 to 1.0 mg by changing the concentration of PFMMA/DOL solution (Table S3). As shown in the voltage profiles of the PFMMA‐coated prelithiated electrodes in Figure S10a, the ICE decreases with increasing PFMMA mass loading, suggesting that higher polymer loading delays the initial activation of the polymer‐coated interface. When evaluated after a fixed resting time of 2 h, anodes with PFMMA mass loading of 0.05 and 0.1 mg achieve ICE as high as 101.8% and 100.5%, respectively. Surface SEM images (Figure S11) further demonstrate that a polymer loading of 0.1 mg yields a uniform and continuous coating across the preSiOC surface, whereas a lower loading of 0.05 mg fails to achieve complete coverage. Consequently, the 0.1 mg polymer‐coated electrode was selected for further analysis. Cross‐sectional SEM images (Figure 1f,g) distinctly reveal the well‐defined layered structure of the 0.1 mg preSiOC/PFMMA electrode, which exhibits a thickness of approximately 540 nm of PFMMA. Complementary EDS mapping confirms homogeneous fluorine distribution within the copolymer layer (Figure 1g). The loading‐dependent decrease in ICE can be further understood from the perspective of interfacial activation. Under an identical resting time, excessive PFMMA coverage may slow the polymer–electrolyte interaction/dissolution process, resulting in less sufficient release and electrochemical utilization of the prelithiated capacity and thus a reduced ICE. This interpretation is supported by the initial charge–discharge profiles obtained after different resting times (Figure S10b), where both the delithiation capacity and ICE are lower at shorter resting times but gradually recover as the resting time is prolonged, indicating the importance of sufficient interfacial activation before testing.

Atomic force microscopy (AFM) analysis in Figure S12 illustrates pronounced surface roughness and height variations between SiOC, preSiOC, and preSiOC/PFMMA electrodes. Quantified surface topography analysis reveals substantially diminished arithmetic average roughness (*R*_a_) and root mean square roughness (*R*_q_) values for the preSiOC/PFMMA electrode, thereby establishing PFMMA's efficacy in generating a conformal coating with markedly enhanced surface planarity and morphological homogeneity. For preSiOC electrode, the F 1s spectra exhibit consistent binding energy at 684.5 eV (Li–F) and 687.5 eV (C–F_2_) across surface and bulk regions (500 s etching, Figure S13) originated from uniformly distributed PVDF binder within the electrode [42]. In contrast, preSiOC/PFMMA (Figure 1h) demonstrates identical F 1s signatures (C–F_n,_ n = 1, 2, 3) to pure PFMMA (Figure S14) in the surface region. Crucially, the characteristic Li–F signal (684.5 eV) emerges only upon bulk etching, indicating the effectiveness of the polymer surface coverage [43].

### 2.3 Dissolution Behavior of PFMMA in Electrolyte

**FIGURE 2:**
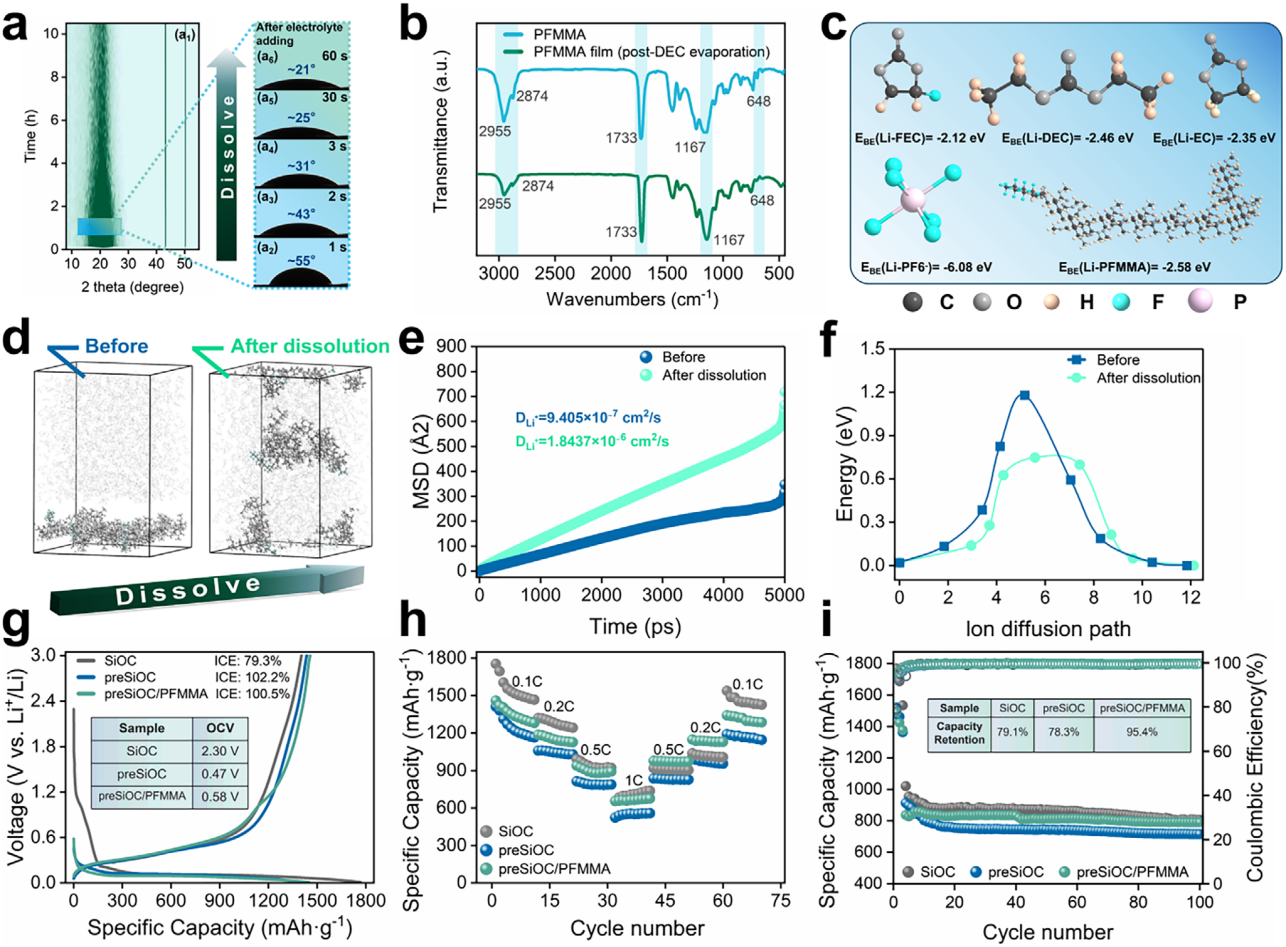
Dissolution behavior and electrochemical properties of PFMMA. (a) In situ XRD patterns of the preSiOC/PFMMA electrode during electrolyte drop‐casting and corresponding contact angle analysis. (b) FTIR spectra of pristine PFMMA and PFMMA film (after DEC evaporation). (c) Calculated binding energies between Li^+^ ions and individual molecular components. (d) Representative MD snapshot of electrolyte structures before/after polymer dissolution. (e) Mean‐squared displacement (MSD) of Li^+^ in the electrolyte with/without polymer dissolution. (f) The calculated energy barrier for Li^+^ migration. (g–i) Electrochemical performance of SiOC, preSiOC, and preSiOC/PFMMA electrodes: (g) initial galvanostatic charge/discharge profiles, (h) rate capability, and (i) long‐term cycling stability at 0.5 C (0.1 C for the first three cycles).

After confirming the uniform coverage of PFMMA across the electrode surface, it is essential to investigate its dynamic dissolution behavior in electrolyte. The solubility of PFMMA copolymer in electrolyte was evaluated through an in situ XRD test for the preSiOC/PFMMA electrode under simulated battery conditions (80 µL, 1.0 m LiPF_6_ in 1:1 w/w EC: DEC, with 5 wt.% FEC). Upon electrolyte immersion, the gradual weakening of this amorphous feature suggests the time‐dependent dissolution of PFMMA. As shown in Figure 2a1, the broad amorphous peak of the preSiOC/PFMMA electrode arises from the combined contribution of the polyimide tape and the polymer (Figure S15). The progressive attenuation of amorphous features upon electrolyte immersion indicates time‐dependent dissolution of PFMMA. Contact angle analysis in Figure 2a2–a_6_ further illustrates the compatibility of PFMMA with the electrolyte: the contact angle of the electrolyte droplet on the PFMMA‐coated electrode decreases from 55° to 25° within 30 s. Significantly, the symmetric cells containing PFMMA in electrolyte exhibit a similar Nyquist plot to that without PFMMA, indicating that the impact of PFMMA coated on ionic conductivity is minimal (Figure S16). In addition, PFMMA was dissolved in an adequate amount of diethyl carbonate (DEC), drop‐cast onto glass, and analyzed by FTIR after complete evaporation of the solvent. As shown in Figure 2b, the reconstituted PFMMA film exhibits identical characteristic peaks to pristine PFMMA, revealing structural integrity upon dissolution‐reconstitution. The surface morphology characterization presented in Figure S17 reveals the formation of a dense and uniform PFMMA polymer coating on the preSiOC surface. Partial polymer dissolution occurs upon the first cycle, as evidenced by the exposed particulate matter from SEM and AFM. Quantitative topographic analysis demonstrates a 230% increase in *R*_q_ (from 177 to 591 nm), signifying electrolyte‐induced surface undulation. Importantly, the absence of morphological defects such as local collapse or cracking validates the preservation of structural integrity. These results collectively suggest that the PFMMA undergoes controlled and homogeneous dissolution in the battery, without compromising the mechanical integrity or inducing structural degradation of the electrode.

Density functional theory (DFT) calculations were performed to evaluate the adsorption energies (*E*_ads_) between Li^+^ and various coordinating species, providing preliminary insights into the possible interaction between PFMMA and Li^+^ and its influence on local Li^+^ transport behavior. As shown in Figure 2c, PFMMA possesses a slightly more negative *E*_ads_ value than conventional carbonate solvents (i.e., DEC, EC, FEC) and a significantly more positive *E*_ads_ value than PF_6_^−^, suggesting that the polar groups in PFMMA are energetically capable of interacting with Li^+^ without introducing an obvious penalty to Li^+^ transport (Figure S18a). Figure 2d illustrates the molecular dynamics (MD) simulation results of a biolayer system composed of bulk electrolyte (the upper layer) and preassembled PFMMA film (the lower layer). After energy minimization, the system was equilibrated under an isothermal‐isobaric (NPT) ensemble (constant number of particles, pressure, and temperature), followed by canonical (NVT) ensemble simulations (constant number of particles, volume, and temperature). As the simulation progressed, PFMMA chains gradually diffused into the electrolyte phase, indicating good compatibility between PFMMA and the carbonate‐based electrolyte.

This compatibility is further supported by Raman and NMR measurements (Figure S19). After PFMMA addition, the Raman spectrum of the electrolyte shows detectable changes: most bands remain at similar positions, but the low‐wavenumber band exhibits a measurable shift from 724.49 to 721.51 cm^−1^. In the ^19^F NMR spectra, the dominant PF_6_^−^ doublet at approximately −73– −76 ppm is still present, yet a clear shift is observed; meanwhile, the FEC‐related signal in the −120–−125 ppm region also displays noticeable changes in peak shape and distribution. These spectral evolutions may reflect minor perturbation of the local fluorine‐containing environment and/or signal overlap involving FEC and the fluorinated side chains of PFMMA. In the ^7^Li NMR spectra, a small but reproducible downfield shift of about 0.01 ppm (from 0.91 to 0.92 ppm) is observed. Taken together, these results demonstrate that PFMMA does not drastically reconstruct the main bulk electrolyte framework, but it does induce weak yet detectable local perturbations in the electrolyte environment. At equilibrium, the Li^+^ self‐diffusion coefficient moderately increases from 9.4050 × 10^−7^ cm^2^·s^−1^ in the pristine electrolyte to 1.8437 × 10^−6^ cm^2^·s^−1^ in PFMMA‐containing system. Based on the ion transport models before/after PFMMA dissolution (Figure S18b), the corresponding Li^+^ migration energy barriers were quantitatively evaluated. As depicted in Figure 2f, the polymer‐modified system exhibits a marginally reduced energy barrier compared to pristine electrolytes. This trend aligns with the results from galvanostatic intermittent titration technique (GITT) measurements (Figure S20). Collectively, these results suggest that PFMMA does not adversely affect Li^+^ transport and may contribute to slightly improved ion‐transport kinetics by maintaining electrolyte compatibility and weakly modulating the local polymer/electrolyte microenvironment.

### 2.4 Electrochemical Performance of PreSiOC/PFMMA Electrode

Electrochemical performance comparisons were conducted to evaluate the influence of surface passivation on electrodes. Figure 2g shows the first galvanostatic charge/discharge (GCD) curves of pristine SiOC, preSiOC, and preSiOC/PFMMA electrodes. The pristine SiOC delivers a relatively low ICE of 79.3% with a substantial irreversible capacity loss (359 mAh·g^−1^) during the first cycle. Prelithiation significantly enhances the ICE of the preSiOC (102.2%) while reducing the open‐circuit voltage (OCV) from 2.30 to 0.47 V. After subsequent surface passivation, the preSiOC/PFMMA anode exhibits an OCV of 0.58 V and maintains a high ICE of 100.5%, indicating that the PFMMA coating barely affects the prelithiation efficiency. The shoulder observed in the delithiation curve of preSiOC/PFMMA may reflect the interfacial regulation effect of the PFMMA coating. The differences between pristine SiOC and preSiOC/PFMMA are also evident in their cyclic voltammetry (CV) curves, in which the SiOC anode displays an additional reduction peak near 0.8 V, attributable to SEI formation (Figure S21). According to the electrochemical impedance spectroscopy (EIS) results (Figure S22 and Table S4), the preSiOC/PFMMA anode exhibits a relatively low overall interfacial resistance (*R_interface_*, 71.03 Ω), much lower than that of pristine SiOC (292.70 Ω). Meanwhile, unlike preSiOC, which shows two distinguishable interfacial processes with separable *R_SEI_* and *Rct*, preSiOC/PFMMA displays only one dominant semicircle, suggesting a simplified and more homogeneous interfacial response after PFMMA coating. Together with the DFT results, these findings suggest that the PFMMA layer does not hinder interfacial charge transfer and may help establish a more compatible electrode/electrolyte interface. Rate performance assessment obtained after excluding the first three activation cycles (Figure 2h, which presents the discharge/lithiation capacity) demonstrates specific capacities of 1356.7, 1168.7, 921.6, and 654.2 mAh·g^−1^ for preSiOC/PFMMA at 0.1 C, 0.2 C, 0.5 C, and 1 C, respectively (The theoretical specific capacity of SiOC is 1800 mAh·g^−1^, for preSiOC and preSiOC/PFMMA, a nominal capacity of 1500 mAh·g^−^^1^ was adopted for C‐rate normalization, rather than as a rigorously calculated theoretical specific capacity of the prelithiated composite electrodes). In comparison, the preSiOC anode delivers lower specific capacities of 1259.0, 1059.2, 817.2, and 572.5 mAh·g^−1^ at the corresponding current densities. Additionally, the preSiOC/PFMMA anode displays a delithiation capacity of 791.4 mAh·g^−1^ with 95.4% retention after 100 cycles at 0.5 C, outperforming the SiOC (79.1% retention) and preSiOC (78.3% retention) electrodes (Figure 2i); its stability is on par with the values reported in the literature.

### 2.5 Ambient Stability of PreSiOC/PFMMA Electrode

**FIGURE 3:**
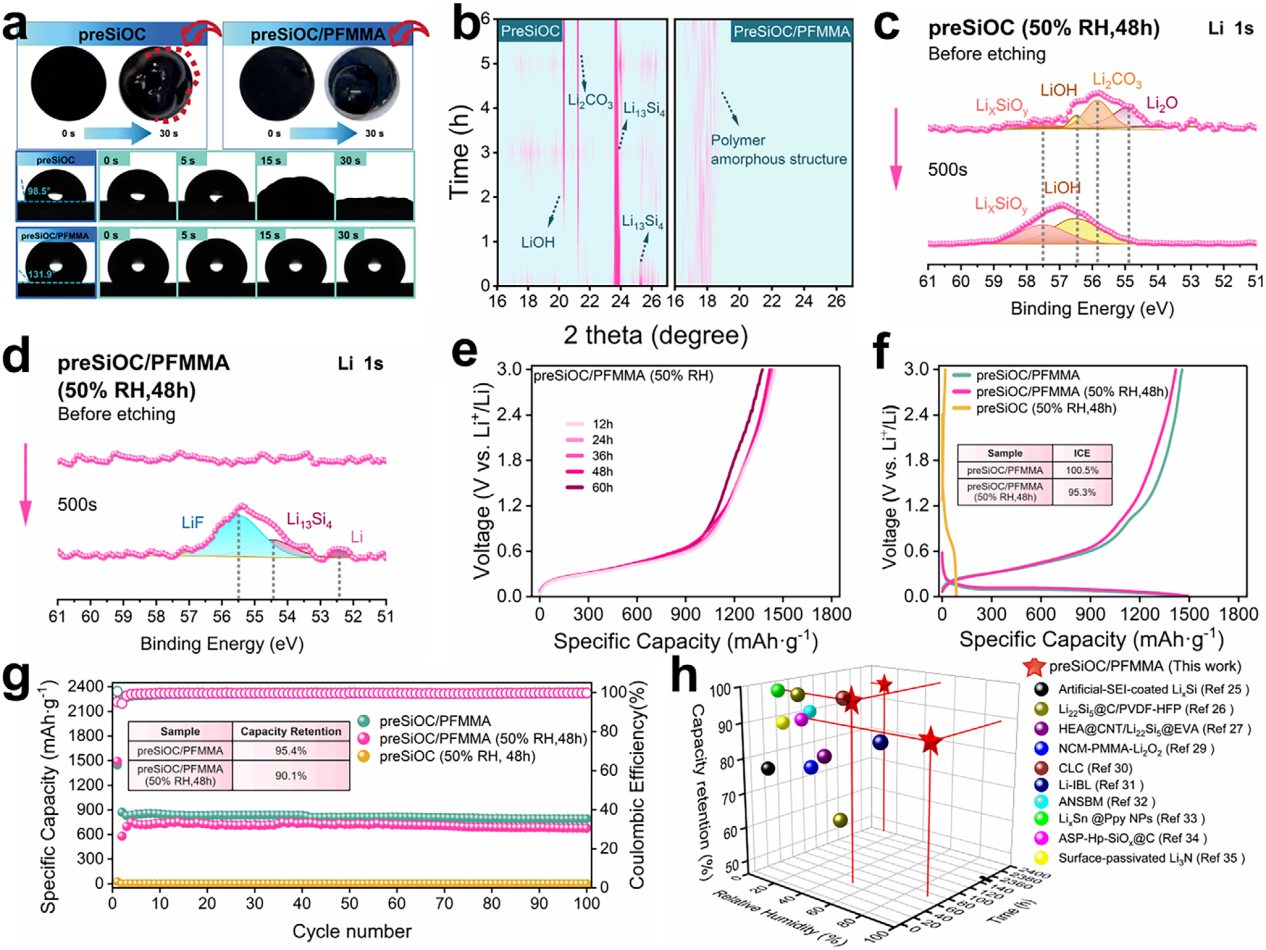
Hydrophobicity and protective performance of the PFMMA coating. (a) Static water contact angles of preSiOC and preSiOC/PFMMA electrodes. (b) In‐situ XRD patterns of electrodes under ambient exposure (∼50% RH). (c,d) XPS spectra of electrodes after 48 h of ambient exposure (∼50% RH). (e) Capacity retention of preSiOC/PFMMA electrodes following different durations of ambient exposure (∼50% RH). (f) Initial galvanostatic charge/discharge profiles of preSiOC and preSiOC/PFMMA electrodes (befor/after exposure). (g) Cycling stability of electrodes at 0.5 C before/after air exposure (∼50% RH, 48 h, 0.1 C for the first three cycles). (h) Comparison of this work with previously reported protection strategies for prelithiated electrodes and prelithiation reagents.

Having established enhanced electrochemical performance attributable to surface passivation, we turn to the practical concern of the electrode's stability under ambient conditions. As shown in Figure 3a, after 30 s of wetting, the contact angle of the water droplet on preSiOC gradually decreases from 98.5° to ∼0° (fully spreading on the electrode surface), accompanied by bubble generation, which should be attributed to the reaction between lithium‐silicon alloy and water. In contrast, the water contact angle of the preSiOC/PFMMA electrode remains nearly constant (from 131.9° to∼130°). After testing, the preSiOC electrode exhibits pronounced deformation, whereas the preSiOC/PFMMA electrode remains structural integrity (Figure S23). The observed contrast indicates that the PFMMA coating effectively suppresses water penetration, thereby forming a protective barrier essential for enhancing the stability of prelithiated electrodes. In situ XRD analysis performed under humid air exposure conditions (Figure 3b) provides further evidence of the improved environmental stability conferred by the PFMMA protective layer. For the unprotected preSiOC electrode, notable formation of lithium hydroxide (LiOH) is observed after merely 30 min of exposure to ambient air. In contrast, the preSiOC/PFMMA electrode shows no detectable crystalline lithium compounds, with diffraction signals corresponding solely to the amorphous polymer, indicating excellent structural stability in humid conditions. The absence of distinct Li_13_Si_4_ peaks in preSiOC/PFMMA does not necessarily indicate its complete disappearance. Although the Cu current collector peaks remain clearly visible because of their strong diffraction intensity, the weak Li–Si alloy reflections can be masked by the increased diffuse scattering background from the amorphous polymer‐coated electrode, making Li_13_Si_4_ difficult to resolve when its crystallinity or content is limited [44, 45].

Environmental resistance was evaluated by aging samples at 50% RH for 48 h followed by XPS depth profiling. The Li 1s spectrum of the pristine preSiOC electrode shows a dominant Li‐Si alloy peak at 54.5 eV (Figure S24a). In contrast, the preSiOC electrode exposed to 50% RH for 48 h displays significant surface chemical transformation with the emergence of Li_2_O (54.9 eV), Li_2_CO_3_ (55.8 eV), LiOH (56.4 eV), and Li_x_SiO_y_ (57.5 eV), confirming parasitic reactions with ambient H_2_O/O_2_ (Figure 3c) [46]. Consistently, the F 1s spectra of uncoated preSiOC also show evident chemical evolution after humid exposure, including shifts of the surface F‐containing components from 684.5/687.5 to 685.0/688.3 eV and the formation of a broad mixed fluorinated component at 685.7 eV after etching, suggesting reconstruction of Li–F‐rich and PVDF‐derived C–F species (Figure S24b). Notably, no surface lithium species are detected on the preSiOC/PFMMA electrode under identical exposure conditions, indicating that the polymer layer can effectively isolate the active materials from environmental reactants (Figure 3d). Post‐etching analysis reveals signals of metallic lithium (52.4 eV), Li_x_Si_y_ alloy (54.5 eV), and LiF (55.5 eV), confirming that the prelithiated electrode retains its structural integrity without evidence of environmental degradation. In contrast to uncoated preSiOC, the F 1s spectra of preSiOC/PFMMA remain nearly unchanged before and after humid exposure, both at the outer surface and after sputtering, indicating that the Li–F‐rich interfacial species and organic C–F components from PFMMA/PVDF are largely preserved (Figure S24c).

Moreover, Figure 3e substantiates the protective efficacy of the PFMMA coating by tracking the evolution of extractable lithium capacity during ambient air exposure (50% RH, 48 h). The preSiOC/PFMMA electrode exhibits an initial lithium extraction capacity of 1457.2 mAh·g^−1^ under inert conditions (Figure 2g). Upon exposure to ambient air (50% RH) for 12, 24, 36, 48, and 60 h, the capacities decreased progressively to 1452.5, 1446.3, 1429.6, 1418.7, 1368.7 mAh·g^−1^, corresponding to 99.7%, 99.3%, 98.1%, 97.4% and 93.9% of the initial capacity, respectively (Figure S25). This gradual decay, attributable to reactions between active materials and moisture/oxygen, confirms PFMMA's effectiveness in delaying degradation within 48 h. Comparative analysis after 48 h of exposure reveals that the unprotected electrode suffered severe performance degradation, primarily due to contamination by uniformly distributed Li_13_Si_4_ alloys. As a result, the cell capacity declined drastically to below 20 mAh·g^−1^ (Figure 3f). In contrast, the PFMMA‐coated electrode preserves significantly improved stability, retaining 90.1% of its capacity after 100 cycles (Figure 3g). Furthermore, the environmental stability of preSiOC/PFMMA electrodes was comprehensively evaluated under harsh conditions: long‐term storage at 10% RH and severe moisture stress at 90% RH. After storing at 10% RH for 100 days, the preSiOC/PFMMA electrodes retain 94.5% of their capacity and subsequently maintain 88.6% of ICE with a capacity retention of 82.9% over 100 cycles (Figure S26a,b). Remarkably, even after 72 h of exposure to 90% RH, the electrode still preserves 88.7% of its capacity and delivers 80.7% of ICE with 74.2% capacity retention over 100 cycles (Figure S26c,d). These results confirm the high effectiveness of the PFMMA layer in enhancing ambient stability under moderate humidity, while also indicating that tolerance to prolonged exposure under extremely high humidity still requires further improvement. In contrast, the TFOA‐0 coated control electrode undergoes pronounced degradation even at 10% RH, further confirming that the incorporation of fluorinated moieties is critical for endowing the protective layer with effective long‐term moisture resistance (Figure S27). Compared to previously reported prelithiated electrode protection strategies, the present work demonstrates record‐setting performance in terms of preservation duration and stability in humid conditions (Figure 3h and Table S5) [24, 25, 26, 27, 28, 29, 30, 31, 32, 33, 34, 35].

### 2.6 Characterization of Post‐Cycled Electrodes

**FIGURE 4:**
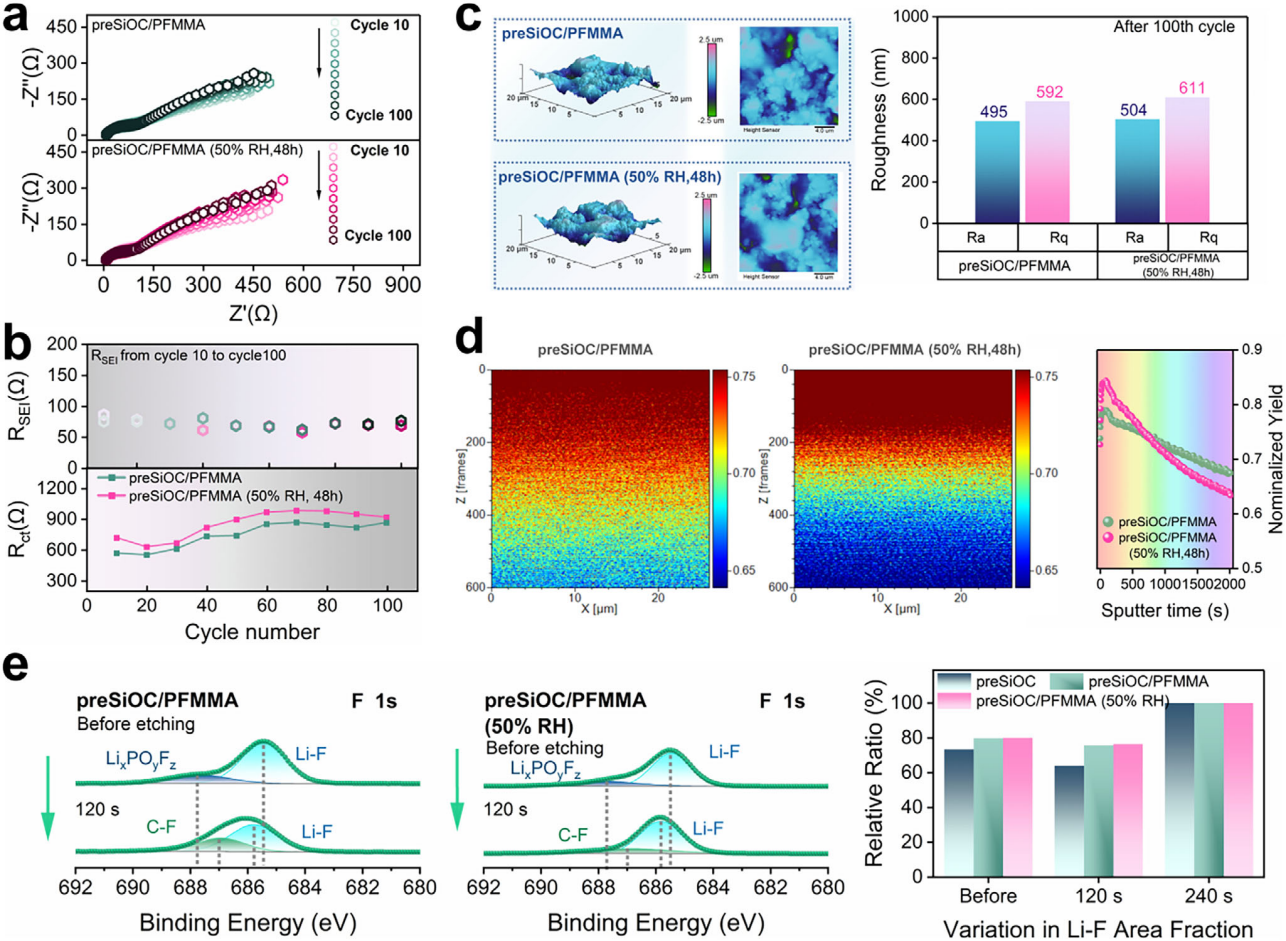
Electrochemical characteristics and interfacial evolution of electrodes upon cycling. (a) Nyquist plots of preSiOC/PFMMA and preSiOC/PFMMA (∼50% RH, 48 h) electrodes, recorded every 10 cycles over 100 discharge cycles. (b) Evolution of charge transfer resistance (*R_ct_*) and SEI resistance (*R_SEI_*) extracted from equivalent circuit fitting of EIS spectra. (c) AFM topography images of electrodes after cycling. (d) TOF‐SIMS analysis of preSiOC/PFMMA and preSiOC/PFMMA (∼50% RH, 48 h) electrodes: spatial distribution of F^−^ species and normalized depth profiles of characteristic ion fragments. (e) XPS spectra of F 1s for electrodes after cycling.

Building on the observations in the previous section, the PFMMA coating demonstrates superior environmental stability, which motivates a detailed investigation of its impact on electrochemical cycling to provide a more comprehensive evaluation of its protective function. Figure 4a shows the EIS measurements of electrodes at 10‐cycle intervals during 100 charge/discharge cycles. The difference in EIS features before and after cycling reflects the evolution of the interface. Before cycling (Figure S22a), the interphase‐related process and the charge‐transfer process are highly overlapped, and therefore only one dominant semicircle is observed. After cycling, with the formation of the cycled interphase, these two processes become more distinguishable, giving rise to two clearly resolved semicircles. As shown in Figure 4b and Figure S28, and Tables S6–S8, the 50% RH‐stored preSiOC/PFMMA electrode exhibits similar resistance values (i.e., ohmic resistance (*R*_Ω_), *R_ct_*, SEI resistance (*R*_SEI_)) and evolution trend similar to those of pristine preSiOC/PFMMA, demonstrating that polymer‐modified interfaces retain electrochemical stability post‐humidity exposure. Surface morphology and roughness of post‐cycled electrodes were analyzed by AFM, as depicted in Figure 4c. The *R*_a_ and *R*_q_ values of the post‐cycled preSiOC/PFMMA electrode are measured to be 495 and 592 nm, respectively, which are higher than those of the preSiOC/PFMMA electrode after the first cycle (Figure S17) due to the progressive dissolution of the PFMMA coating polymer and the volume expansion nature of SiOC. Notably, the preSiOC/PFMMA electrode stored at 50% RH for 48 h after cycling shows only a marginal increase of approximately 2%–3% in both *R_a_* and *R*_q_ compared with the non‐humidity‐treated counterpart, underscoring the polymer's protective efficacy against structural and performance degradation under humidity. Time‐of‐flight secondary ion mass spectrometry (TOF‐SIMS) depth profiling in Figure 4d reveals similar fluorine distribution for the post‐cycled preSiOC/PFMMA and preSiOC/PFMMA (50% RH, 48 h) electrodes. Dark red regions in the depth profile maps indicate fluorine enrichment extending into the bulk region for PFMMA‐loaded electrodes (preSiOC/PFMMA and preSiOC/PFMMA (50% RH, 48 h)). Quantitative analysis reveals that both electrodes exhibit similar fluorine contents, distinctly higher than those of the unmodified samples (Figure S29). The humid air‐exposed sample also exhibits a slightly steeper fluorine‐depth gradient, suggesting a somewhat more heterogeneous through‐thickness distribution of fluorine‐containing species. Depth‐resolved fluorine profiling further confirms that the PFMMA‐modified electrodes possess a broader spatial distribution and higher concentration of fluorine species.

The observed compositional changes are associated with the evolution of SEI at the electrode–electrolyte interface [47]. High‐resolution XPS spectra (Figure 4e and Figures S30 and S31) confirm the presence of typical SEI constituents in all three electrodes, including Li_2_CO_3_/ROCO_2_Li species (C 1s ∼290.2 eV) and LiF (Li 1s ∼56.7 eV after etching) [48, 49, 50]. In polymer‐modified electrodes, O 1s shows O─C═O features at the surface (∼534.0 eV), which may arise in part from residual polymer segments redeposited during sample preparation. For the polymer‐modified electrodes (preSiOC/PFMMA, with/without humid exposure), the Li 1s peak at the surface (Li 1s ∼55.9 eV) is shifted by about +0.4 eV to higher binding energy compared with pristine preSiOC (Li 1s ∼55.5 eV). This shift reflects a lower electron density around surface Li atoms, attributable to the strong electronegativity of C–F groups in the polymer, and it may reflect an increased contribution of Li–O–rich inorganic phases in the inner SEI induced by the polymer coating. In the F 1s spectra, all electrodes display a feature at ∼687.8 eV before etching, corresponding to Li_x_PO_y_F_z_ species from LiPF_6_ decomposition (Figures 4e and Figure S29) [51]. After 120 s of etching, the LiF fraction decreases to ∼65% in preSiOC but remains higher (∼76%) in PFMMA‐modified electrodes. Notably, the LiF peak shifts from ∼685.2 to ∼685.5 eV upon polymer coating, reflecting a more electron‐withdrawing environment. This shift indicates that the presence of the polymer promotes stronger F^−^–Li^+^ interactions, thereby enhancing the chemical stability of SEI [52]. As shown in Figure S30, the C 1s spectra support this interpretation: the unmodified electrodes exhibit strong C─O (∼286.5 eV at the surface) and O─C═O peaks (∼290.2 eV at the surface), while in the PFMMA‐modified electrodes these electrolyte decomposition products/organic carbonates (Li_2_CO_3_/ROCO_2_Li) related peaks are significantly attenuated, indicating suppression of organic decomposition products. Overall, the PFMMA coating contributes to the development of a stable and homogeneous SEI by enriching LiF in the surface layer and mitigating the formation of organic side products, while also demonstrating excellent stability under humid exposure.

### 2.7 Electrochemical Assessment of Lithium‐Ion Full Cell

**FIGURE 5:**
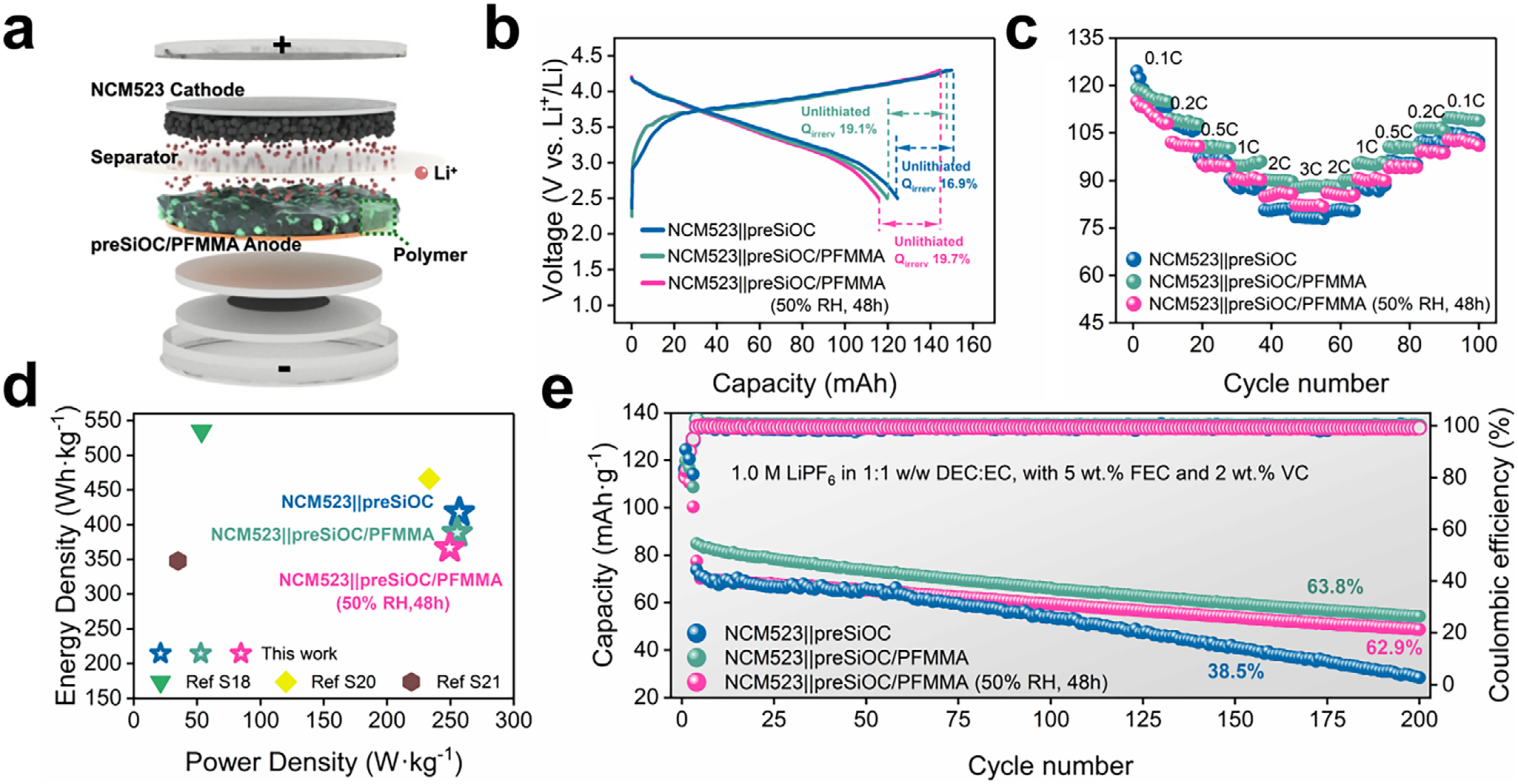
Electrochemical performance of full‐cell configurations. (a) Schematic illustration of the coin‐type full‐cell configuration, with polymer layer positions indicated. (b) Comparison of the initial coulombic efficiency among NCM523||preSiOC, NCM523||preSiOC/PFMMA, and NCM523||preSiOC/PFMMA (50% RH). (c) Rate capability at various current densities. (d) Ragone plot in comparison with energy densities at power densities from previous literature. (e) Long‐term cycling stability and coulombic efficiency over prolonged operation at 0.5 C (0.1C for the first three cycles).

To evaluate the practical applicability of the interfacial prelithiation layer under realistic operational conditions, coin‐type lithium‐ion full cells were assembled by pairing preSiOC, preSiOC/PFMMA, and preSiOC/PFMMA (50% RH) anodes with LiNi_0.5_Co_0.2_Mn_0.3_O_2_ (NCM523) cathode, and underwent electrochemical characterization in a voltage range of 2.75–4.20 V, the N/P ratio was determined to be ∼1.05 (Table S9). Notably, the preSiOC electrode stored at 50% RH for 48 h had already been demonstrated to fail in half‐cell tests, therefore, it was not further included in the subsequent full‐cell discussion. Figure 5a shows the position of the polymer in the battery installation. As shown in the initial charge–discharge curves in Figure 5b and Figure S32, the NCM523||SiOC full cell exhibits an ICE of 65.4%, whereas the NCM523||preSiOC full cell exhibits an ICE of 83.1%. By contrast, the NCM523||preSiOC/PFMMA configuration shows a marginal ICE reduction to 80.9%. This slight decrease in ICE is likely associated with the initial dissolution and interfacial adjustment of the PFMMA layer, during which a small fraction of the prelithiated lithium may not be fully released. Noteworthy, the NCM523||preSiOC/PFMMA (50%RH, 48h) full cell retains an ICE of 80.3%, demonstrating exceptional electrochemical activity preservation under humid storage conditions, which is attributable to the superior protective efficacy of the PFMMA layer. Rate capability tests show that the NCM523||preSiOC/PFMMA full cell after humid storage (50% RH, 48 h) delivers 116.9, 107.7, 99.3, 93.7, 89.2, and 88.3 mAh·g^−1^ at 0.1, 0.2, 0.5, 1, 2, and 3 C, respectively. The performance is comparable to that of the NCM523||preSiOC/PFMMA pristine cell (88.9 and 83.9 mAh·g^−1^ at 1C and 3 C, respectively) (Figure 5c), demonstrating that the PFMMA interphase preserves interfacial kinetics and electrochemical activity after humid exposure.

Energy and power densities were calculated based on the total mass of the active materials and the PFMMA polymer (Table S10). The Ragone plots in Figure 5d benchmark the three full‐cell configurations against reported silicon‐anode systems. The pristine NCM523||preSiOC/PFMMA cell delivers an energy density of 417.9 Wh·kg^−1^, highlighting the benefit of prelithiation. After humid storage (50% RH, 48 h), the corresponding cell retains an energy density closely comparable to the pristine case, underscoring the effectiveness of the PFMMA interphase in preserving prelithiation‐enabled advantages under humid conditions. Figure 5e summarizes the long‐term cycling performance. After storage at 50% RH for 48 h, the NCM523||preSiOC/PFMMA full cell retains 62.9% of its initial capacity after 200 cycles, on par with the 63.8% retention of the pristine NCM523||preSiOC/PFMMA cell, yet substantially higher than the 38.5% retention of the unmodified NCM523||preSiOC system. The difference indicates that the PFMMA interphase effectively maintains cycling stability and preserves the prelithiation‐enabled electrochemical activity following humid exposure. Importantly, the resulting cycling stability is comparable to representative SiOC‐anode‐based full‐cell systems reported in the literature [53, 54].

## 3 Conclusions

For the first time, a fluorine‐rich acrylate copolymer (PFMMA) was rationally designed, synthesized, and implemented to address a central bottleneck in prelithiation, the pronounced air‐ and humidity‐sensitivity of prelithiated electrodes. It is demonstrated that the strategic incorporation of abundant fluorinated moieties and methyl methacrylate units endowed the PFMMA with distinguished hydrophobicity and electrolyte compatibility, thus achieving a critical balance between ambient stability and electrochemical functionality. Consequently, the PFMMA‐modified preSiOC electrode delivers remarkable environmental adaptability with unprecedented retained capacity and ICE under various humidity conditions, including 10% RH, 50% RH, and 90% RH. Full‐cell validation further confirms PFMMA's efficacy in preserving prelithiation benefits under operational conditions. The study validates the efficacy of molecularly engineered copolymer coatings in constructing moisture‐resistant interfaces, enabling practical handling during manufacturing while ensuring electrochemical performance parity with pristine prelithiated systems.

## Author Contributions

**Ruifeng Zheng**: data curation. **Rong Chen**: formal analysis, data curation. **Pingshan Jia**: formal analysis. **Huaiyu Shao**: conceptualization, funding acquisition, writing – review and editing, supervision. **Yingying Shen**: formal analysis. **Yixuan Fan**: data curation, formal analysis. **Yun Zheng**: data curation, formal analysis. **Luojiang Zhang**: conceptualization, writing – review and editing, supervision. **Congcong Zhang**: data curation, formal analysis. **Yongbing Tang**: conceptualization, writing – review and editing, funding acquisition, supervision, resources. **Yinan Liu**: data curation, formal analysis.

## Conflicts of Interest

The authors declare no conflicts of interest.

## Supporting information




**Supporting File**: advs76562‐sup‐0001‐SuppMat.docx.

## Data Availability

The data that support the findings of this study are available in the supplementary material of this article.
