# Supplementary material for: Targeted Multifunctional Fluorine‐Rich Copolymer Coating Design for Ambient‐Stable Prelithiated SiOC Anodes
Source: Adv Sci (Weinh). 2026 Aug 3:e76562. Online ahead of print. doi: 10.1002/advs.76562 (PMC13430929; doi:10.1002/advs.76562)
Supplement: Supplementary file 1 — Supporting File: advs76562‐sup‐0001‐SuppMat.docx. [file ADVS-9999-e76562-s001.docx]

DOI: 10.1002/ ((please add manuscript number))

**Article type:** **Full Paper**

**Targeted Multifunctional Fluorine-Rich Copolymer Coating Design for Ambient-Stable Prelithiated SiOC Anodes**

*Rong Chen*^†^*, Yixuan Fan, Congcong Zhang, Yinan Liu, Yun Zheng, Ruifeng Zheng, Yingying Shen, Pingshan Jia, Luojiang Zhang,* Yongbing Tang,* Huaiyu Shao**

1. Chen, C.C. Zhang, Y.N. Liu, R.F. Zheng, Dr.Y. Zheng, Y.Y. Shen, P.S. Jia, Prof. H.Y. Shao

Institute of Applied Physics and Materials Engineering, University of Macau, Avenida da Universidade, Macao, SAR, 999078, China

R. Chen, Y.X. Fan, Prof. L.J. Zhang, Prof. Y.B. Tang

Advanced Energy Storage Technology Research Center, Shenzhen Institute of Advanced Technology, Chinese Academy of Sciences, Shenzhen 518055, China

Prof. Y.B. Tang

University of Chinese Academy of Sciences, Beijing 100049, China

***Corresponding author.** E-mail: [hshao@um.edu.mo,](mailto:hshao@um.edu.mo,) [tangyb@siat.ac.cn](mailto:tangyb@siat.ac.cn), [zhanglj@siat.ac.cn](mailto:zhanglj@siat.ac.cn)

**Experimental section**

**Material preparation**

Butyl acetate was purchased from Guangzhou Chemical Reagent Factory. Azobisisobutyronitrile (AIBN), methyl methacrylate (MMA, CAS: 80-62-6), and 1,3–dioxolane (DOL, CAS: 646-06-0) were obtained from Shanghai Macklin Biochemical Co., Ltd. 3,3,4,4,5,5,6,6,7,7,8,8,8–Tridecafluorooctyl methacrylate (TFOA, CAS: 2144-53-8) was supplied by Shanghai Aladdin Biochemical Technology Co., Ltd. Conductive carbon (Super P), separators (PP), copper foil, and other battery components (including cell cases and gaskets et.al) were all purchased from Shenzhen Kejing Star Technology Company. Electrolyte (1.0 M LiPF_6_ in 1:1 w/w DEC:EC, with 5 wt.% FEC for half-cells and 1.0 M LiPF_6_ in 1:1 w/w DEC:EC, with 5 wt.% FEC and 2 wt.% VC for full cell) were provided by Dongguan Kelude Experimental Equipment Technology Co., Ltd. The cathode material LiNi_0.5_Co_0.2_Mn_0.3_O_2_ (NCM523) was provided by Gotion High-tech Co., Ltd. Poly (vinylidene fluoride) (PVDF, HSV900) was provided by Arkema. A commercial silicon oxide composite (SiOC) material was sourced from Hong Kong AOLISIN Technology Limited. Pre-mixed powder containing lithium metal particles and silicon powder was obtained from Shandong Zhongshan Photoelectric Materials Co., Ltd.

**Synthesis of PFMMA**

In this experiment, butyl acetate was the reaction solvent, and AIBN was the initiator for synthesizing fluorine-rich hydrophobic acrylate copolymer *via* free radical solution polymerization. The selected formulation was determined on the basis of preliminary optimization, according to the results of contact angle, surface morphology, and thermal stability tests. The reaction occurred in a four-necked flask with a cantilever stirrer, nitrogen inlet, condenser, and constant pressure funnel. The formulation of PFMMA used in this work was selected based on preliminary optimization of monomer composition, initiator content, and crosslinker dosage, contact angle, surface morphology, and thermal stability. The specific process is as follows: after deoxygenation with nitrogen, butyl acetate (30 g) was added and heated at 90 °C with stirring for 30 minutes (300 r·min^-1^). MMA (10 g) and TFOA (2 g) were mixed with AIBN (0.9 g) and dripped into the flask over 2 hours with stirring (300 r·min^-1^), followed by heating. Remaining AIBN (0.3 g) and additional butyl acetate (5 g) were added, maintaining the temperature for another 2 hours (300 r·min^-1^), and then remove the reactants after cooling. The resulting material was then diluted to 10% mass concentration, coated onto a glass slide, and evaporated at room temperature for 12 hours to obtain fluorinated acrylate copolymer (PFMMA).

**Synthesis of Li_13_Si_4_ particles**

In an argon-filled glove box, 1 g of pre-mixed powder (containing lithium metal particles and silicon powder) and 3 g of Zirconia balls were placed inside a stainless-steel container sealed with an agate milling jar (45 mL). Ball-milling was done in a planetary mill (P7, Fritsch, Germany) for 4 hours at a 400-rpm rotational speed.

**Characterization**

The successful synthesis of the fluorinated acrylate copolymer PFMMA was confirmed by ^19^F NMR spectroscopy (Bruker Avance 400 MHz). Fourier-transform infrared (FTIR) spectra were recorded using FTIR spectrometer (Thermo Fisher Scientific Nicolet iS20). Thermogravimetric analysis (TGA) was performed on the thermogravimetric analyzer (METTLER TOLEDO, TGA2). The surface morphology and cross-sectional microstructure of the electrode were characterized using scanning electron microscopy (SEM; Zeiss Sigma 300). Energy dispersive X-ray spectroscopy (EDS, Bruker Quantax XFlash SDD 6-30) mapping identified pronounced fluorine enrichment within the film structure. The chemical composition was analyzed by X-ray photoelectron spectroscopy (XPS) using a Thermo Scientific ESCALAB Xi^+^ XPS Microprobe system, and at an acceleration voltage of 12 kV and a filament current of 6 mA, with pass energies of 150 eV for survey scans (step size: 1 eV) and 50 eV for high-resolution scans (step size: 0.1 eV). The crystal structure of the synthesized samples was characterized by powder X-ray diffraction (XRD) patterns were collected using a Rigaku Smart Lab diffractometer equipped with Cu Kα radiation (λ = 1.5406 Å), the instrument was operated at 40 kV and 50 mA, with a scanning range of 5° to 80° (2θ). All in-situ XRD measurements in this study were conducted under identical conditions. Atomic force microscopy (AFM, Bruker Dimension Icon) was employed to characterize the surface roughness and topographic profiles of electrodes. Time-of-flight secondary ion mass spectrometry (TOF-SIMS, Carl Zeiss XB540) was employed to analyze the longitudinal distribution of F^-^ ions across electrode sheets.

The hydrophobicity was evaluated through contact angle (θ_CA_) measurements. A 5 μL water droplet was deposited onto the surface and analyzed using a digital microscope (OSA100S-T, Ningbo NB Scientific Instruments Co. Ltd., China), the dynamic behavior on the surface was captured with a high-speed camera (775 fps frame rate, Ningbo Scientific Instruments Co. Ltd., China). The samples were put in substrates and placed on the instrument's testing platform. Ultrapure water was selected as probe liquids; each dispensed at a droplet volume of 5 μL. All values represent average results from at least three independent replicate measurements. The electrode characterization under humid conditions was performed using a commercial humidity detector (DELIXI, THM-01) coupled with a humidifier. The prelithiated electrode was placed in a sealed box to maintain airtight conditions. The relative humidity was precisely adjusted to the target level using the humidifier, after which the humidification process was terminated. Besides, to evaluate the wettability of the electrode surface with respect to the electrolyte, contact angle measurements were conducted under the same conditions as the previous hydrophobicity tests. Specifically, the test liquid was replaced with the electrolyte (1.0 M LiPF_6_ in 1:1 w/w DEC: EC, with 5 wt.% FEC), and the resulting contact angles were recorded to assess the surface affinity toward the electrolyte.

**Electrochemical measurement**

The Li_13_Si_4_ electrodes were fabricated by blending Li_13_Si_4_ particles, Super P, and PVDF (65:15:15 mass ratio) in a DOL solvent. The homogeneous slurry was uniformly coated onto copper foil and dried at 80°C for 12 h, yielding a surface loading about 1 mg·cm^-2^. The entire process was conducted in an argon-filled glove box to ensure an inert atmosphere.

The SiOC electrodes were fabricated by blending SiOC particles, Super P, and PVDF (65:15:15 mass ratio) in DOL solvent. The slurry was uniformly coated onto copper foil and dried at 80°C for 12 h, yielding a surface loading of active material about 1 mg·cm^-2^. The entire process is conducted under ambient atmospheric conditions.

For 20 wt.% preSiOC electrode, SiOC particles, Li_13_Si_4_, Super P, and PVDF (50:20:15:15 mass ratio) were dispersed in DOL. The homogeneous slurry was then cast onto copper foil and vacuum-dried at 80°C for 12 h, maintaining a surface loading of active material about 1 mg·cm^-2^. The preSiOC/PFMMA electrode was prepared by spraying a prelithiated electrode with DOL-dissolved PFMMA copolymer solution (magnetically stirred for 2 h, 300 r·min^-1^, ambient temperature), the PFMMA loading was regulated through polymer concentration and spray volume. The entire process was conducted in an argon-filled glove box to ensure an inert atmosphere. The theoretical specific capacity of SiOC is 1800 mAh·g^-1^ and a nominal capacity of 1500 mAh·g^-1^ for preSiOC and preSiOC/PFMMA electrodes. The latter is a nominal current-normalization value for the prelithiated composite electrode rather than a strict theoretical specific capacity.

Before the test, all coin cells were stored at 25°C for 10 h. The galvanostatic measurement was performed using a battery tester (Neware CT-4008) testing system within a potential range of 0.01-2.0 V (vs. Li^+^/Li). The cyclic voltammetry (CV) and the electrochemical impedance spectroscopy (EIS) data of the CR2032 coin cells were recorded using an electrochemical workstation (Modulab XM system, AMETEK).

**Density functional theory (DFT) calculation**

All calculations were performed based on the DFT [1,2]. The PBE-GGA functional and PAW method are used with the energy cutoff of 520 eV. The Van der Waals (vdW) interactions were considered using DFT-D3 method. Spin polarization is considered. The structure is no longer relaxed when the energy convergence is less than 10^-6^ eV and the force at each atom is less than 0.01 eV·A^-1^. Finally, the adsorption energies (E_ads_) were calculated as E_ads_= E_ad/ion_ -E_ion_ -E_ad_, where E_ad/ion_, E_sub_, and E_ad_ are the total energies of the optimized adsorbate/ion system, optimized ion, and adsorbate with ion, respectively.

The MD simulations for structures of electrolyte systems were performed using the MD software Material Studio. During the simulation processes, the force field was selected as COMPASS III. A cutoff distance of 12.5 Å was applied in the calculation of electrostatic interactions and van der Waals interactions. The systems were equilibrated through a three-step strategy. First, a 5000 step steepest descent method is employed to avoid unreasonable contact. Secondly, 1 ns NVT simulation is carried out at 300 K. Subsequently, we performed 50-ns NPT simulations at 300 K and 1 bar, and the post-30-ns production trajectories were used to calculate radial distribution functions (RDF). Newton’s equations of particle motion were integrated using a leap-frog algorithm with a 2-fs time step. The long-range electrostatic and van der Waals (vdW) interactions calculated up to 13 Å using the particle-mesh Ewald method [3,4]. The velocity-rescaling thermostat and the Berendsen barostat were used to retain the system's temperature and pressure, respectively [5,6]. The calculation of the MSD (mean square displacement) was based on the extraction of the last 5000 ps of the dynamics.


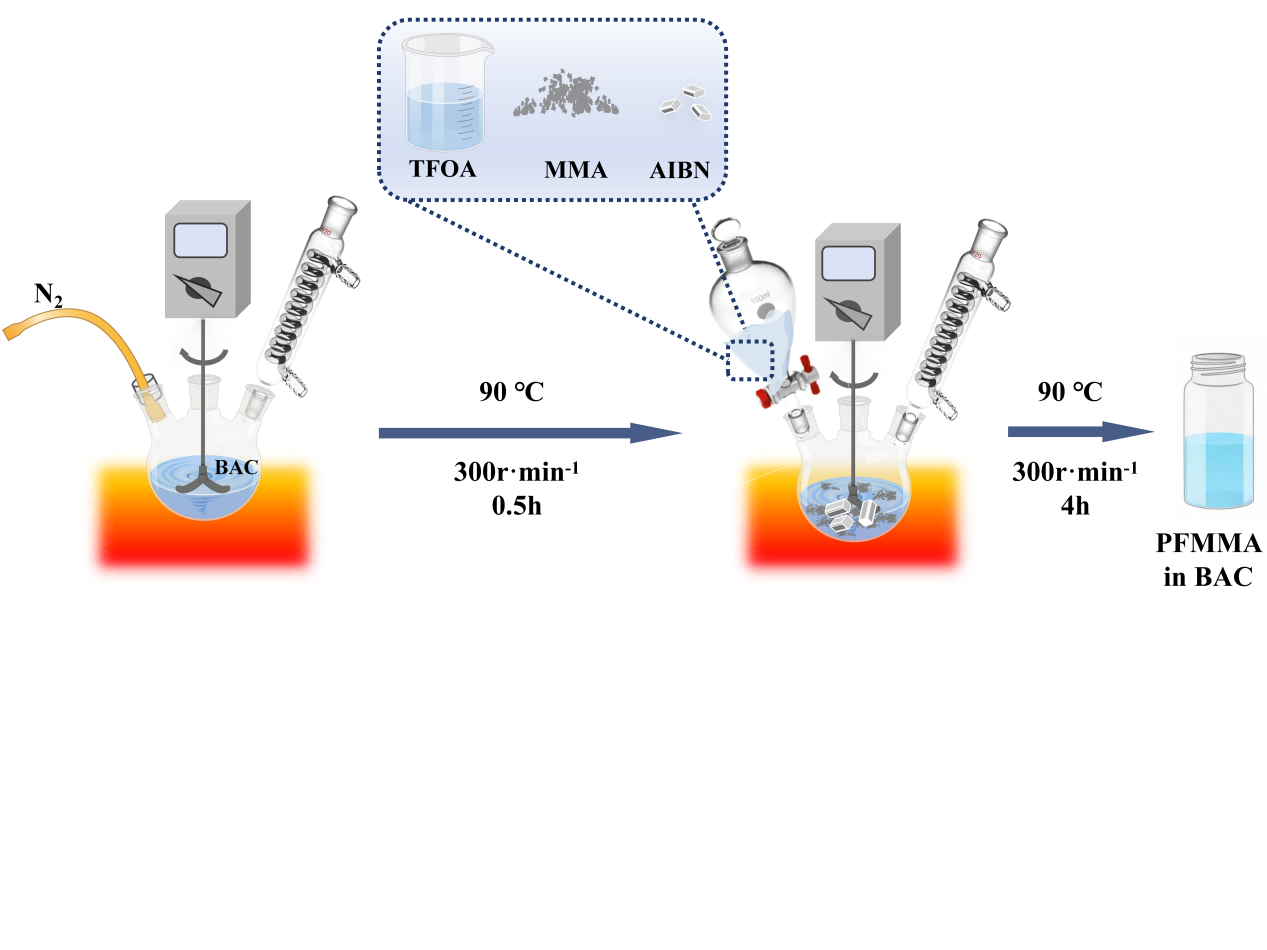


**Figure S1.** The synthetic procedure of PFMMA.


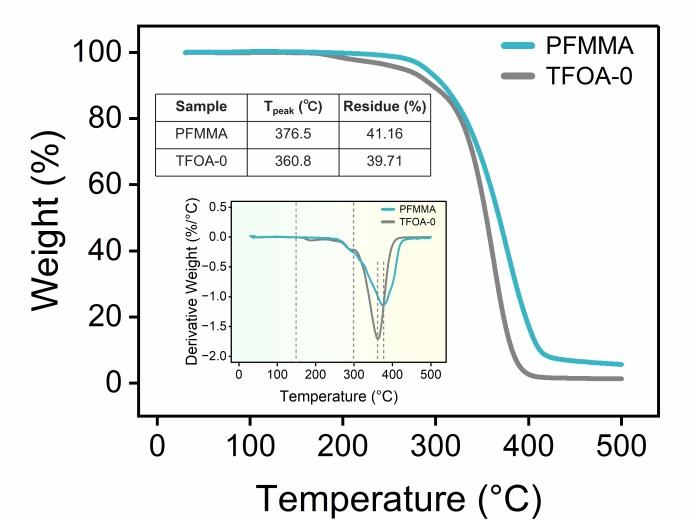


**Figure S2.** TGA curves of PFMMA and TFOA-0.


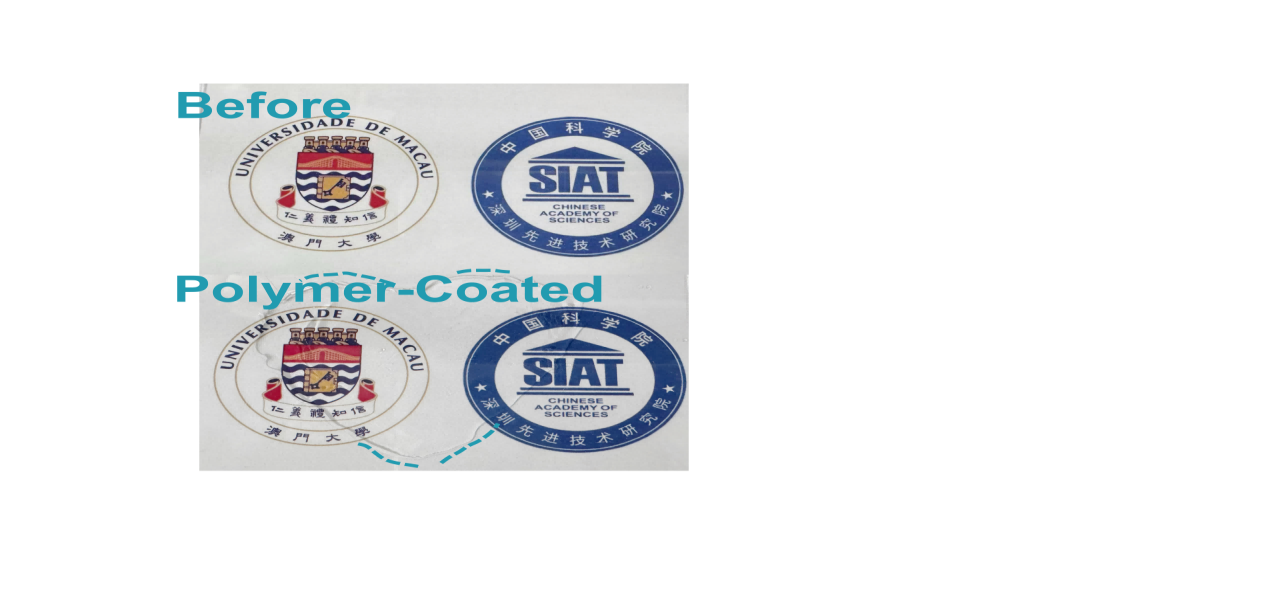


**Figure S3.** The as-prepared PFMMA films are highly transparent with good film-forming characteristics.


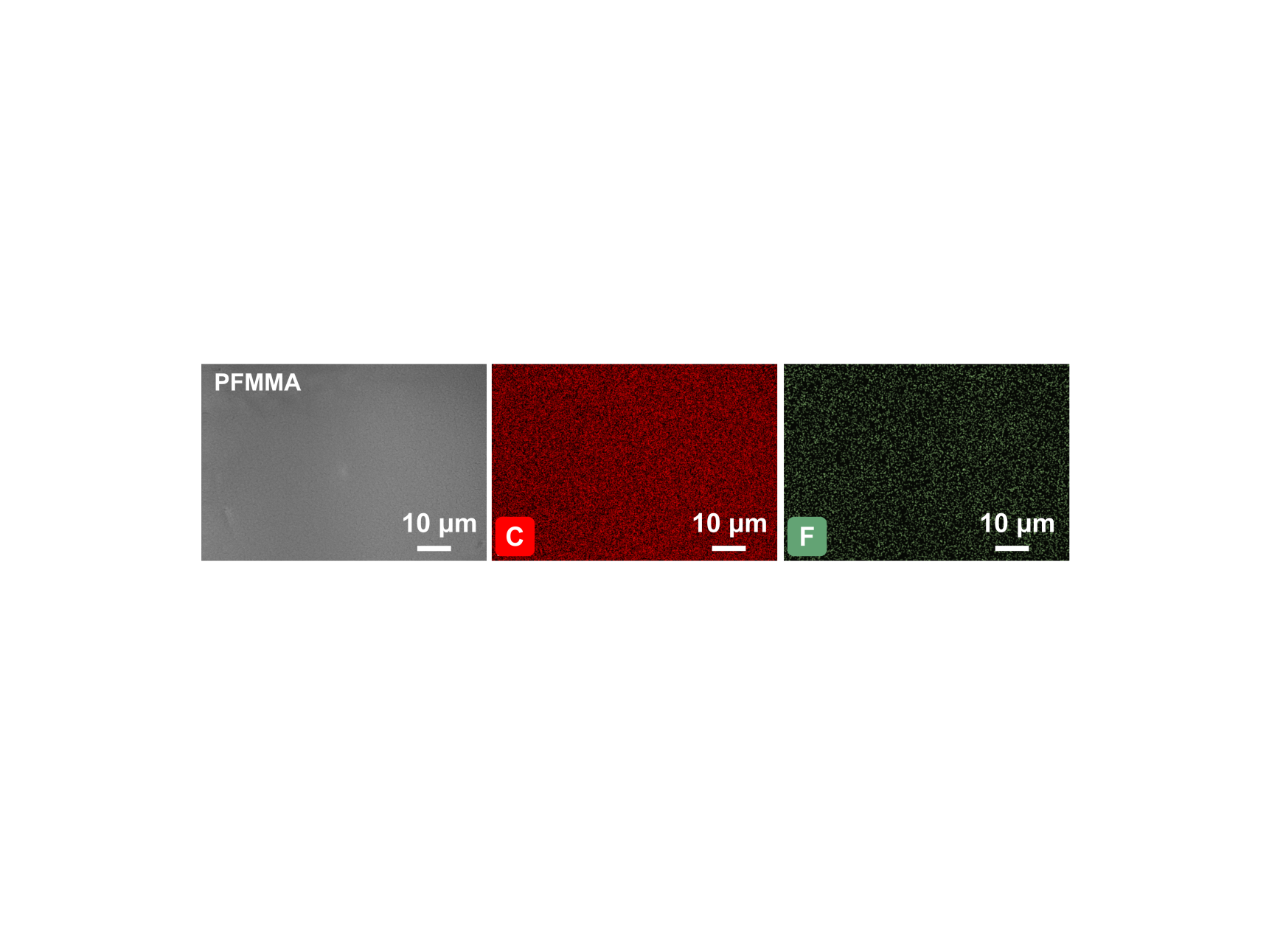


**Figure S4.** SEM images of PFMMA, and the corresponding EDX mappings of C element and F element.

**
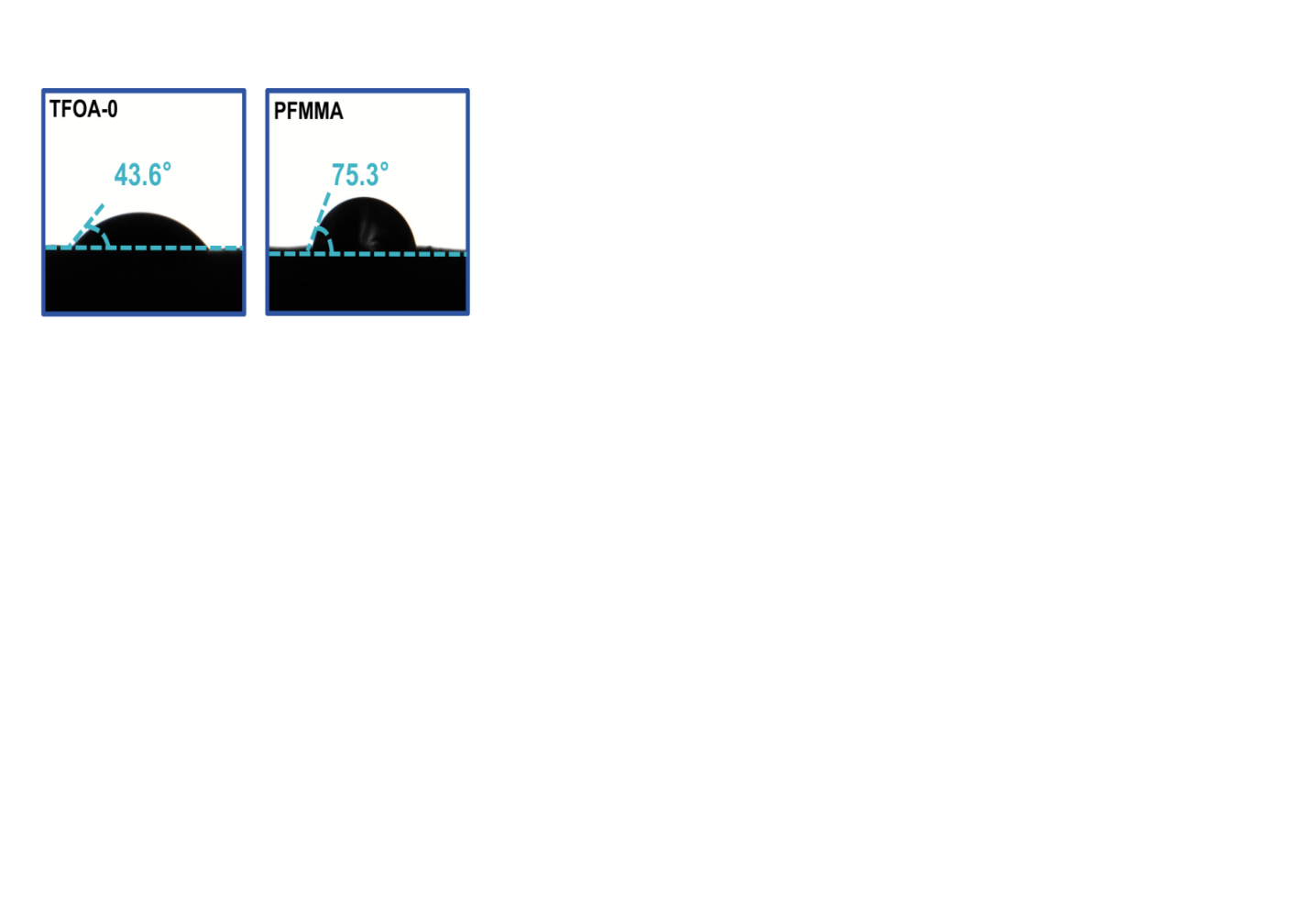
**

**Figure S5.** Contact angles of TFOA-0 and PFMMA using diiodomethane as probe liquids.

**Surface energy calculations.** Surface energy were carried out using the Owens–Wendt two liquid method [7,8], which separates the total surface energy of a solid into its dispersive (non-polar) and polar components. The method is based on the following equations:

$\text{γ}_{\text{S}}\text{=}{\text{γ}_{\text{S}}}^{\text{D}}\text{+}{\text{γ}_{\text{S}}}^{\text{P}}$ (1)

$\text{γ}_{\text{L}}\text{=}{\text{γ}_{\text{L}}}^{\text{D}}\text{+}{\text{γ}_{\text{L}}}^{\text{P}}$ (2)

Here, $\text{γ}_{\text{S}}$ denotes the total surface energy of the solid, ${\text{γ}_{\text{S}}}^{\text{D}}$ the dispersive component, and ${\text{γ}_{\text{S}}}^{\text{P}}$ the polar component. Similarly, $\text{γ}_{\text{L}}$, ${\text{γ}_{\text{L}}}^{\text{D}}$, and ${\text{γ}_{\text{L}}}^{\text{P}}$ represent the total, dispersive, and polar components of the liquid’s surface tension, respectively. The relationship between these parameters and the contact angle is expressed as:

$\text{γ}_{\text{L}}\left（ \text{1+}\text{cosθ} \right）\text{=2(}{{\text{γ}_{\text{S}}}^{\text{D}}{\text{γ}_{\text{L}}}^{\text{D}}\text{)}}^{\frac{\text{1}}{\text{2}}}\text{+2(}{{\text{γ}_{\text{S}}}^{\text{P}}{\text{γ}_{\text{L}}}^{\text{P}}\text{)}}^{\frac{\text{1}}{\text{2}}}$ (3)

In Equation (3), θ is the measured contact angle of the liquid on the solid surface (as shown in Figure S5). Given that the polar and dispersive components of the probe liquids are known, this equation allows for the determination of ${\text{γ}_{\text{S}}}^{\text{D}}$ and ${\text{γ}_{\text{S}}}^{\text{P}}$ by solving a system of two equations derived from two different liquids. Once these components are determined, the total surface energy $\text{γ}_{\text{S}}\text{ }$can be calculated using Equation (1). In this study, ultrapure water was selected as the polar liquid, diiodomethane was selected as the nonpolar liquid with a droplet volume of 5 μL for each measurement, the surface tension components used in the calculations are summarized in Table S1, calculated surface free energys are summarized in Table S2.


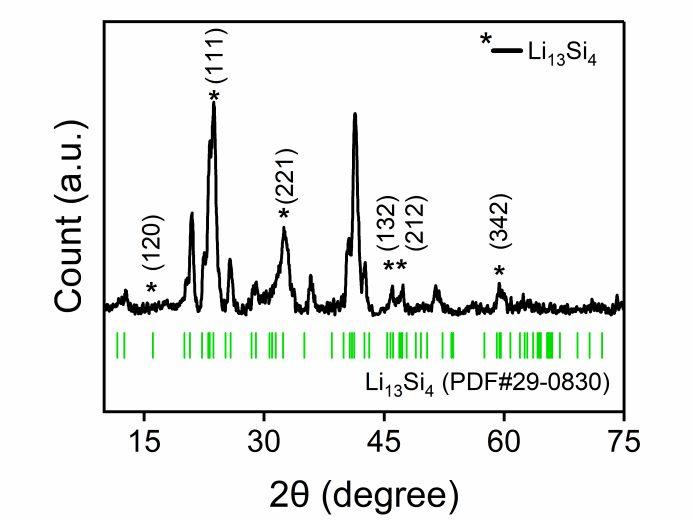


**Figure S6.** XRD characterization of ball-milled prelithiated agent Li_13_Si_4_.


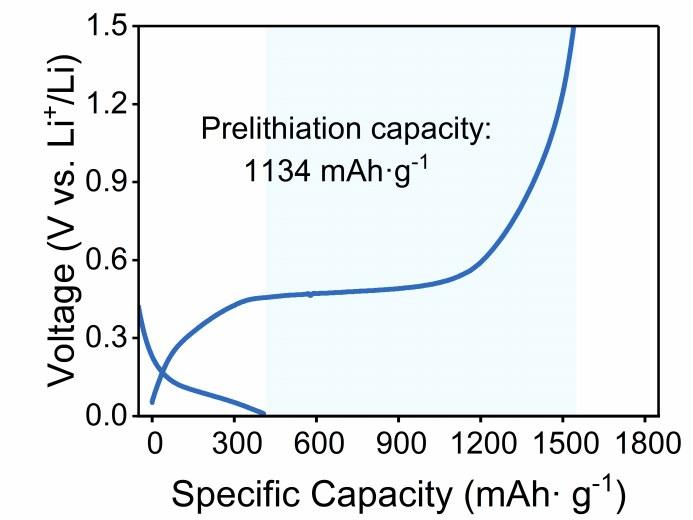


**Figure S7.** Initial charge/discharge profiles of Li_13_Si_4_||Li half-cell.


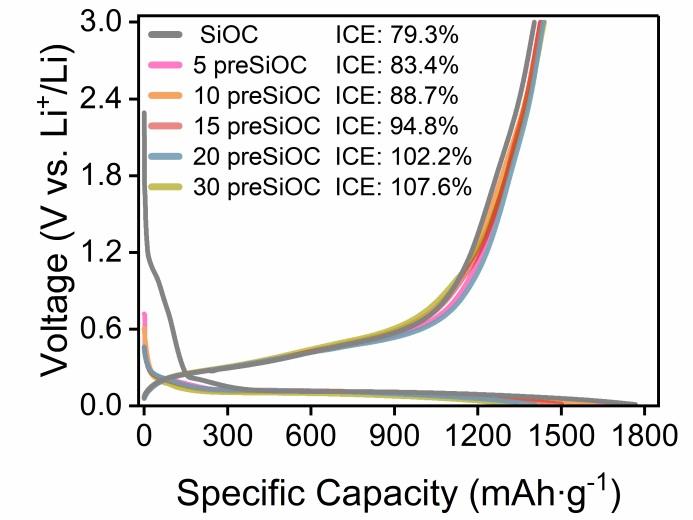


**Figure S8.** Voltage profiles of preSiOC||Li half-cells with varying Li_13_Si_4_ additive amounts.


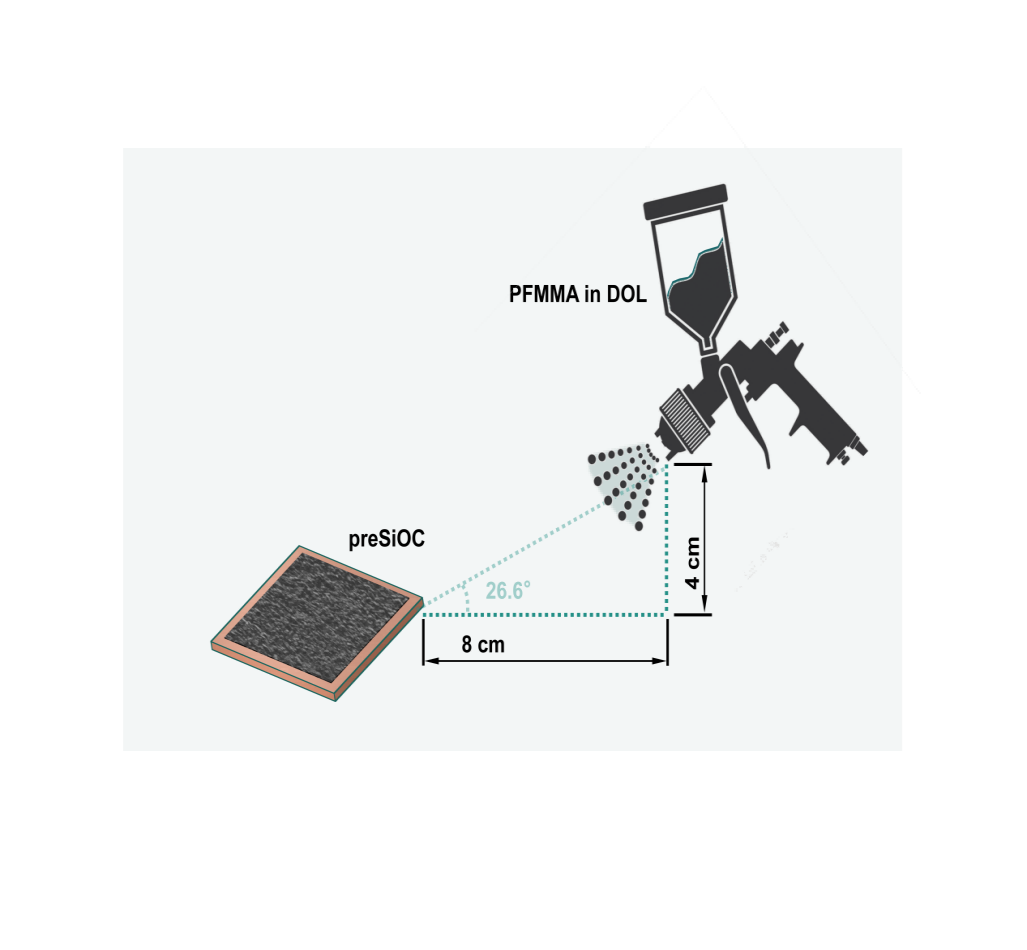


**Figure S9.** Schematic illustration of the PFMMA spray coating setup.


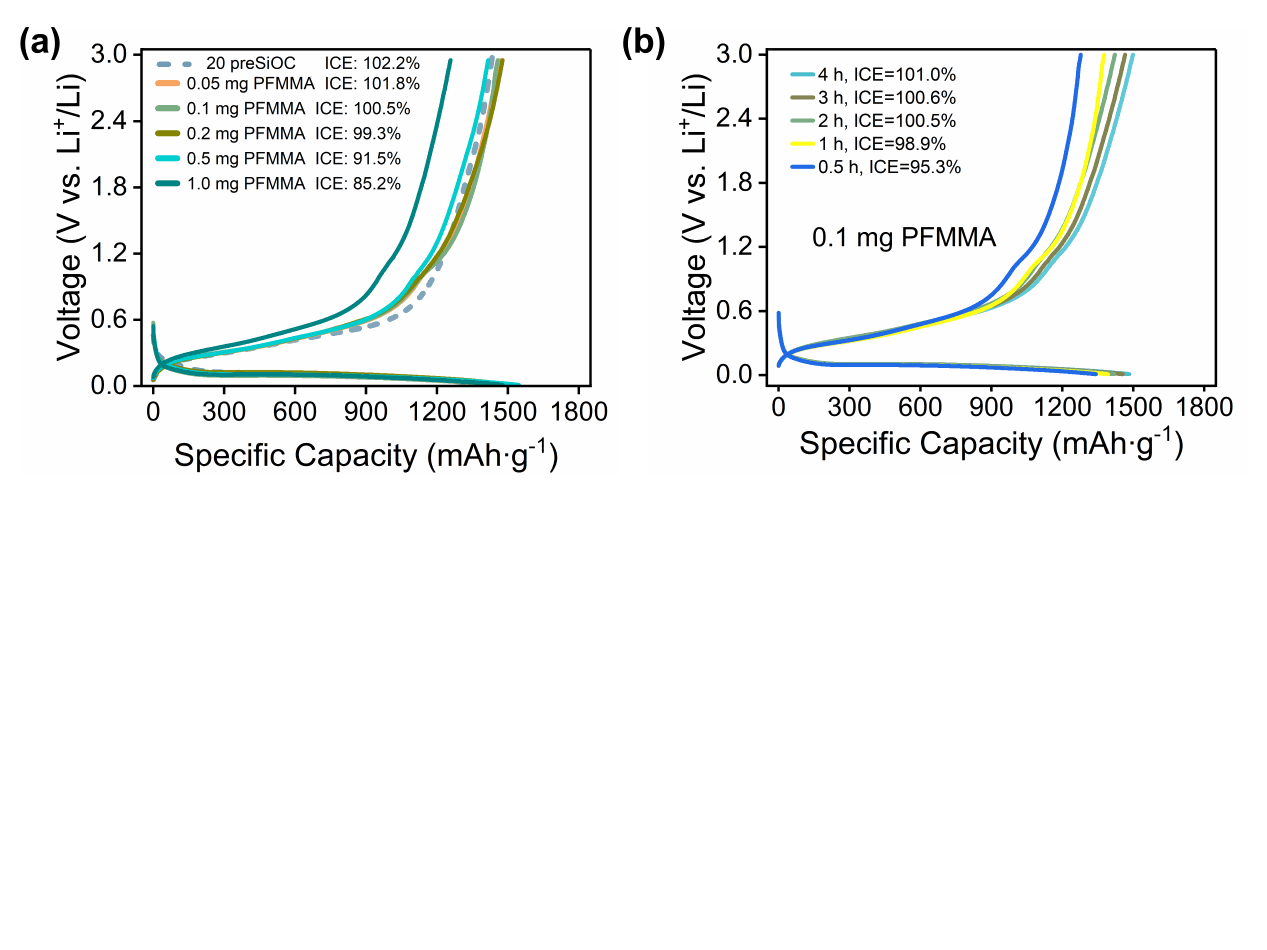


**Figure S10.** Electrochemical performance of preSiOC/PFMMA||Li half-cells with (a) different PFMMA loadings after a 2 h resting period, and (b) different resting times.


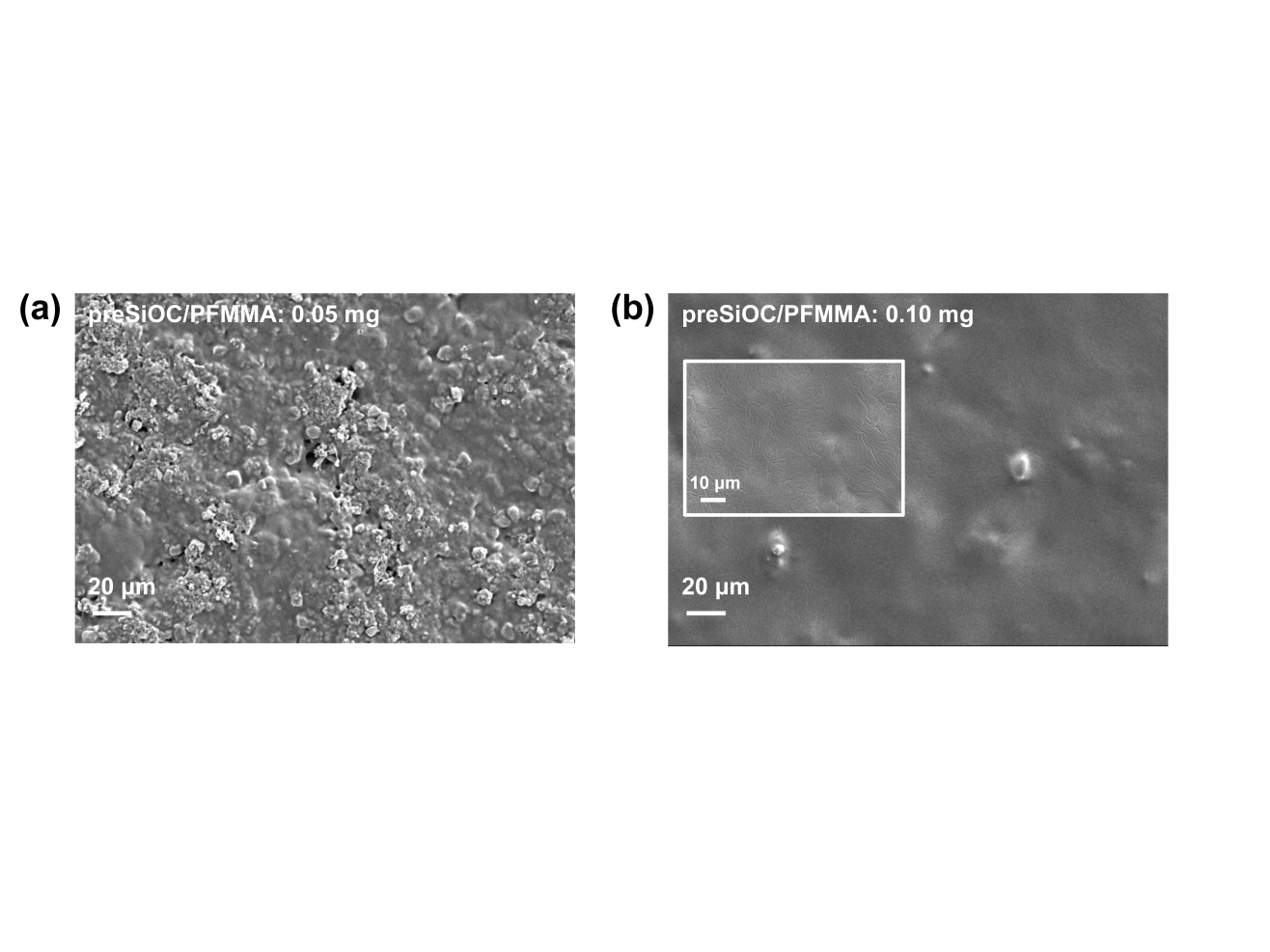


**Figure S11.** SEM images of preSiOC/PFMMA with PFMMA loadings of (a) 0.05 mg and (b) 0.1 mg.


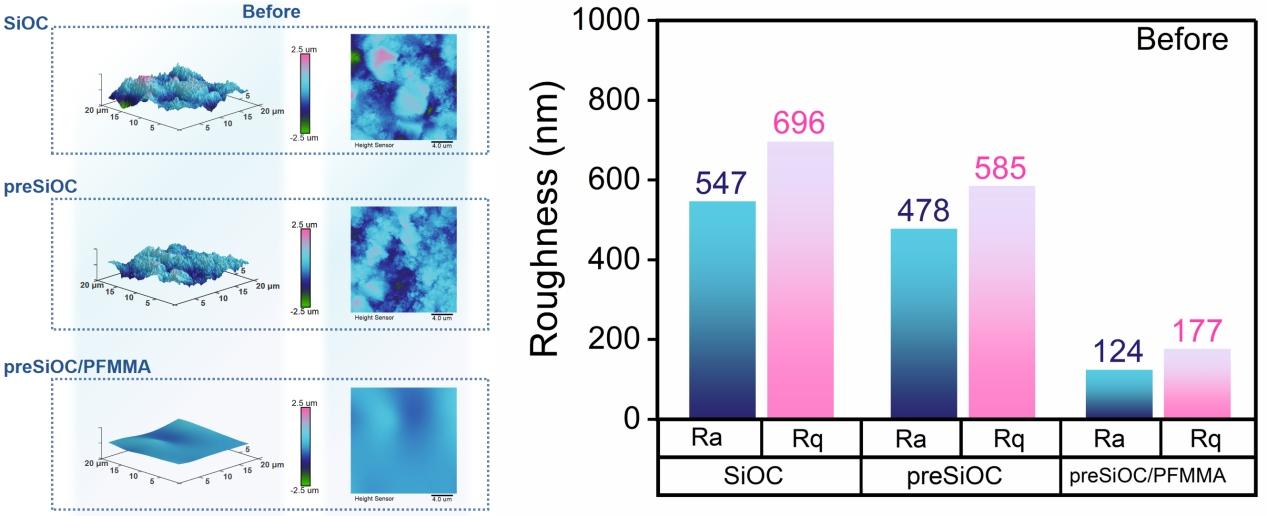


**Figure S12.** 3D AFM topography and surface roughness of SiOC, preSiOC and preSiOC/PFMMA electrodes.

*R*_a_ (Arithmetic Average Roughness) and *R*_q_ (Root Mean Square Roughness) are critical for evaluating electrode-electrolyte interfacial properties in battery electrode characterization:(i) provides a straightforward measure of overall surface irregularity, thereby evaluating active material distribution and coating homogeneity; (ii) Lower *R*_a_/*R*_q_ values typically indicate smoother electrodes, enhancing interfacial contact with separators and reducing inhomogeneous Li^+^ flux.

**Arithmetic Average Roughness (*R*_a_):**

*R*_a_ is defined as the arithmetic mean of the absolute deviations of the surface height from the mean height. It quantifies the average surface roughness over the scanned area and is calculated using the following formula:

$$\text{R}_{\text{a}}\text{=}\frac{\text{1}}{\text{L}}\sum_{\text{i}\text{=1}}^{\text{n}} \text{r}_{\text{i}}\text{l}_{\text{i}}\text{}$$

where *r* is the vertical deviation of the real outline from the benchmark outline, *l* and *L* are the segment length and total the length of the benchmark outline, respectively.

**Root Mean Square Roughness (*R*_q_):**

*R*_q_ represents the root meaning square deviation of surface height values and reflects the degree of height variation across a surface [9]. *R*_q_ is more sensitive to extreme peaks and valleys compared to *R*_a_. The mathematical expression for R_q_ is:

$$\text{R}_{\text{q}}\text{=}\sqrt{\frac{\text{1}}{\text{L}}\sum_{\text{i=1}}^{\text{n}} {\text{r}_{\text{i}}}^{\text{2}}\text{l}_{\text{i}}}$$

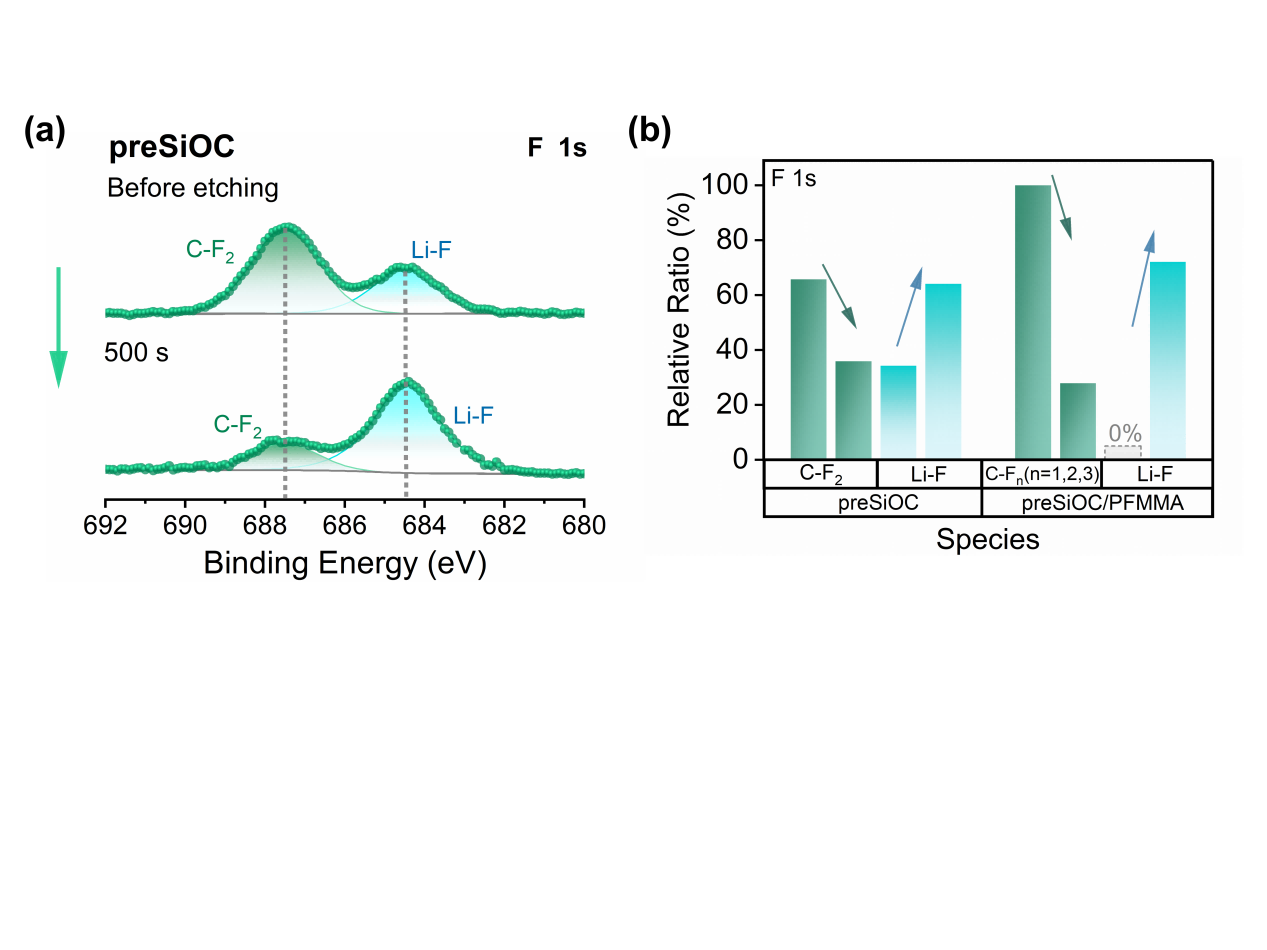


**Figure S13.** (a) XPS depth profiling of F 1s spectra and (b) quantitative analysis of component distribution, comparing preSiOC and preSiOC/PFMMA electrodes.


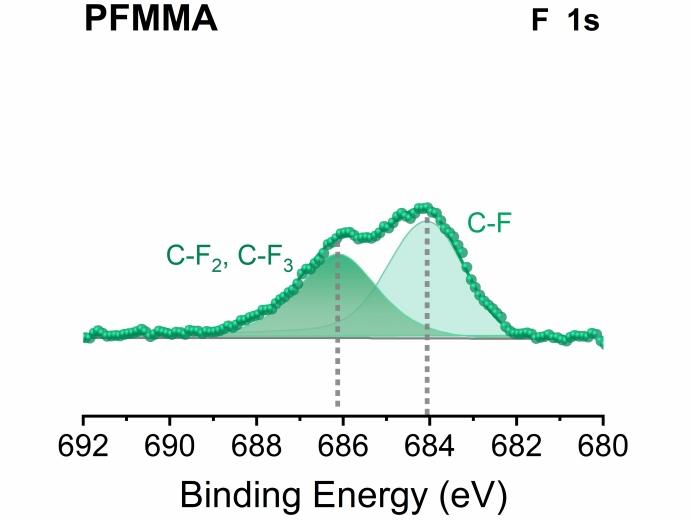


**Figure S14.** XPS spectrum of F 1 s for the PFMMA polymer.

**
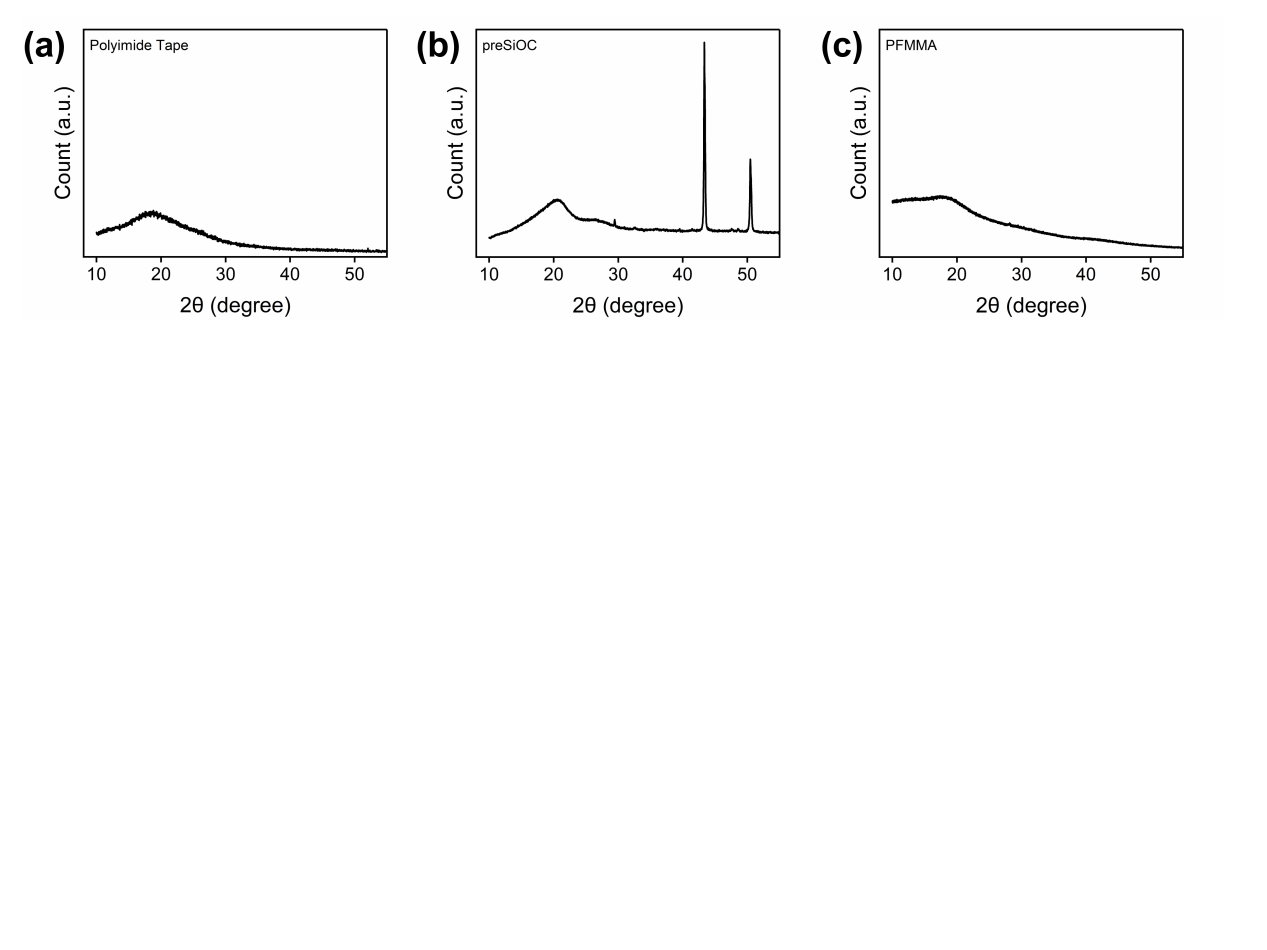
**

**Figure S15.** XRD Characterization of (a) polyimide tape, (b) preSiOC electrode covered with polyimide tape, and (c) pristine PFMMA film without polyimide tape.

As shown in Figure S15 (a), XRD analysis of the polyimide tape reveals a broad amorphous diffraction feature at around 20° to 22°, with no evidence of sharp crystalline peaks. Figure S15 (b) reveals that the preSiOC electrode without polymer loading displays a broad amorphous diffraction feature at ~20° to 22°, which can be attributed to the polyimide tape, while the sharp reflections at ~43.3° and 50.5° originate from the current copper collector. As shown in Figure S15 (c), XRD analysis of the PFMMA polymer reveals its amorphous nature, exhibiting a broad scattering feature at around ~18° to 20° and a lack of sharp crystalline peaks.


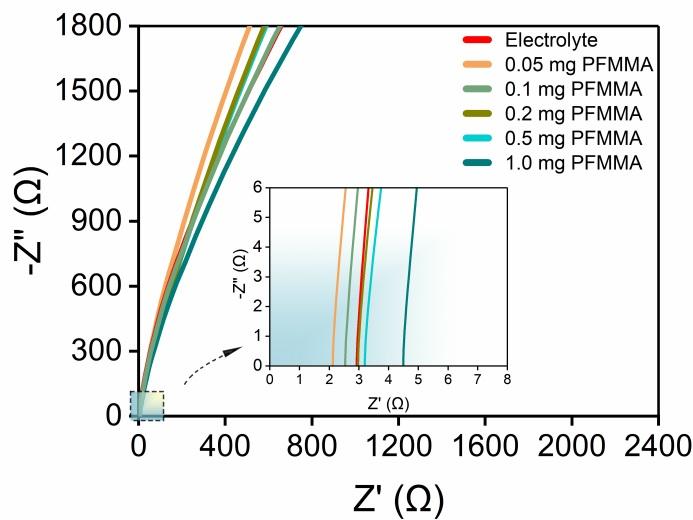


**Figure S16** Nyquist plots of symmetric stainless-steel cells assembled with electrolytes containing varying concentrations of dissolved PFMMA.


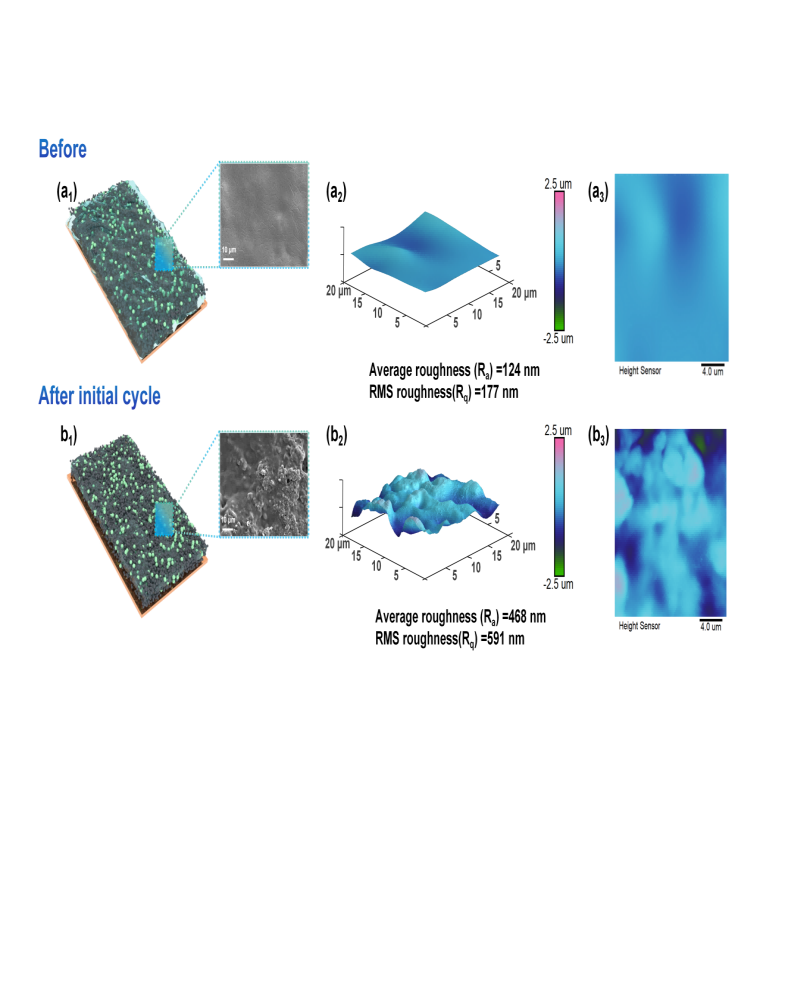


**Figure S17.** Comparative SEM micrographs of the preSiOC/PFMMA electrode surface collected before and after the initial electrochemical cycle, accompanied by AFM characterization of surface morphology and spatial roughness distribution.


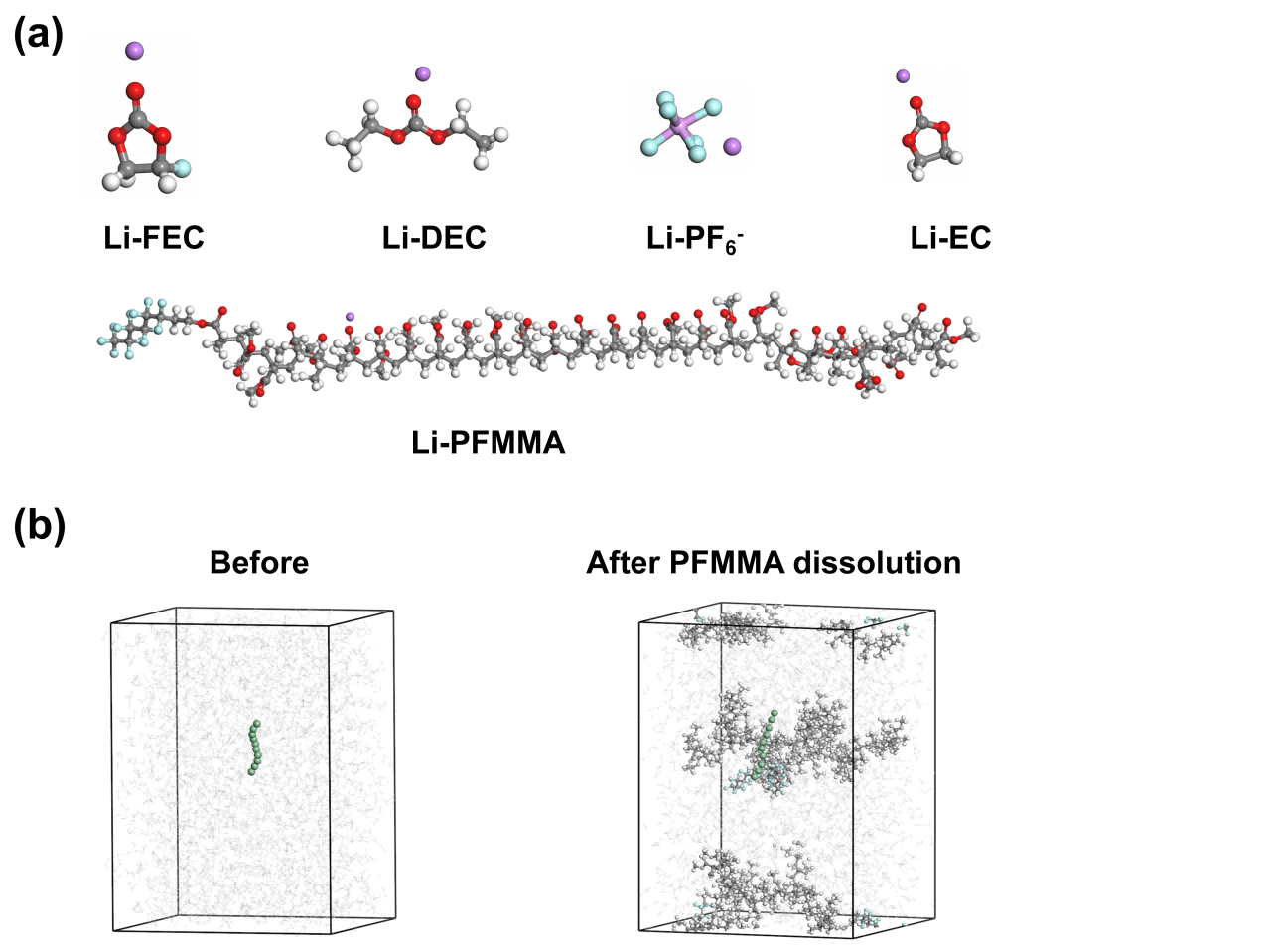


**Figure S18.** (a) Geometry-optimized Li⁺ coordination structures correspond to the binding-energy calculations in Figure 2c. (b) Ion transport model before and after PFMMA dissolution.


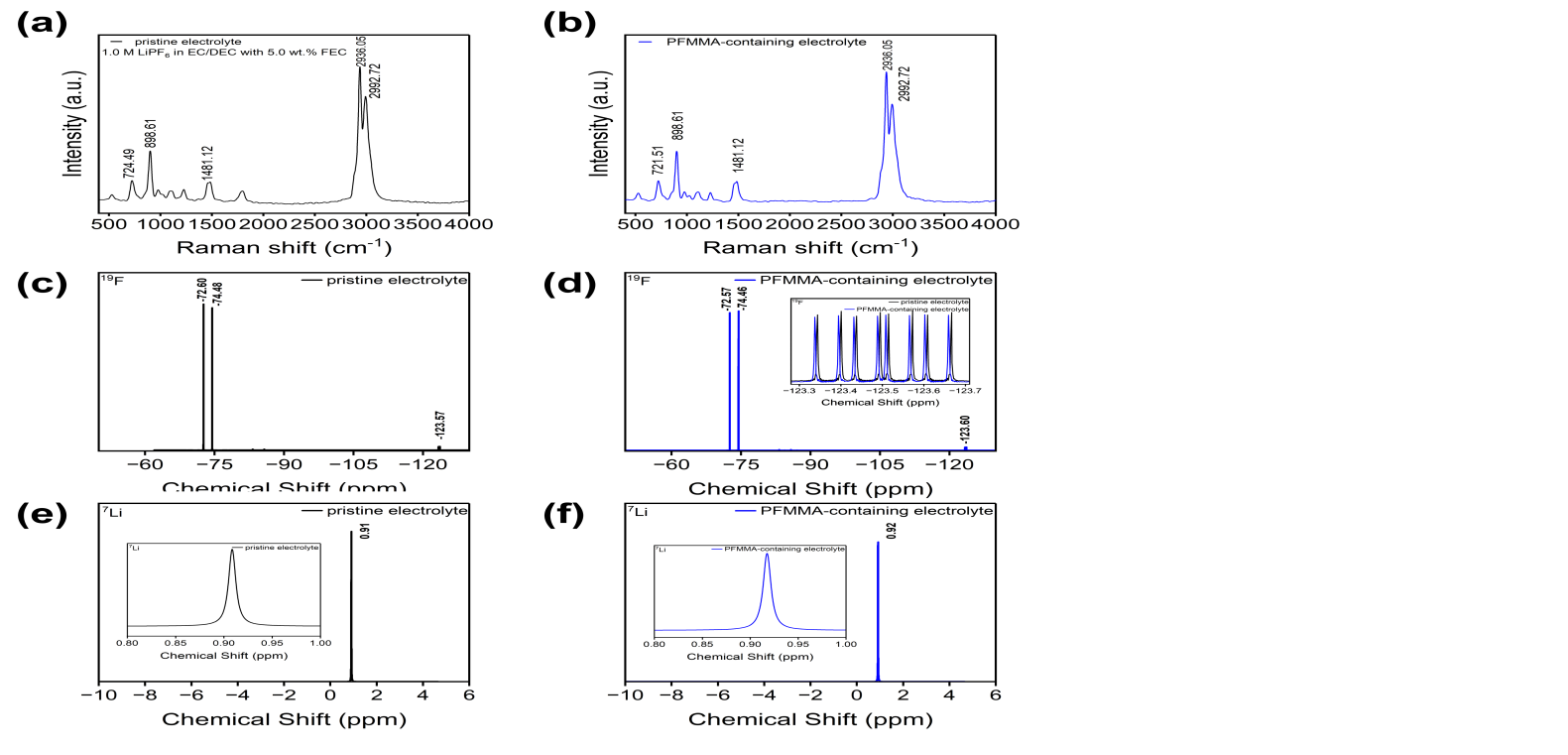


**Figure S19.** (a,b) Raman spectra, (c,d) ^19^F NMR spectra and (e,f) ^7^Li NMR spectra of the pristine electrolyte, 1.0 M LiPF_6_ in EC/DEC (v/v = 1:1) with 5.0% FEC, and the PFMMA-containing electrolyte prepared with 1.0 mg PFMMA in 80 μL electrolyte. Inset in (d): Enlarged ^19^F NMR spectra in the -120 to -125 ppm region, showing the FEC-related fluorine signal before and after PFMMA addition.


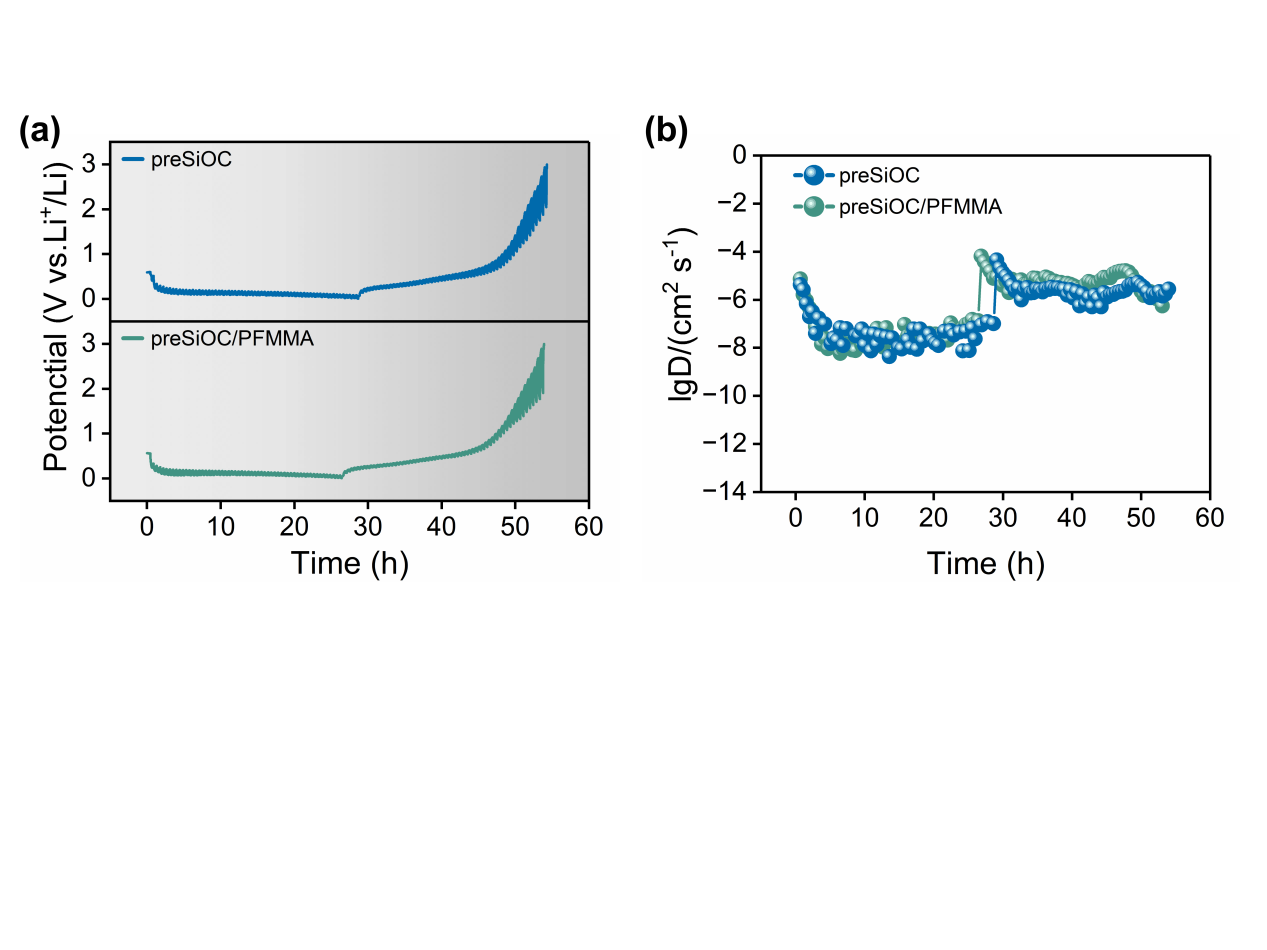


**Figure S20.** (a) GITT profiles and (b) corresponding Li^+^ diffusion coefficient of preSiOC and preSiOC/PFMMA electrodes.


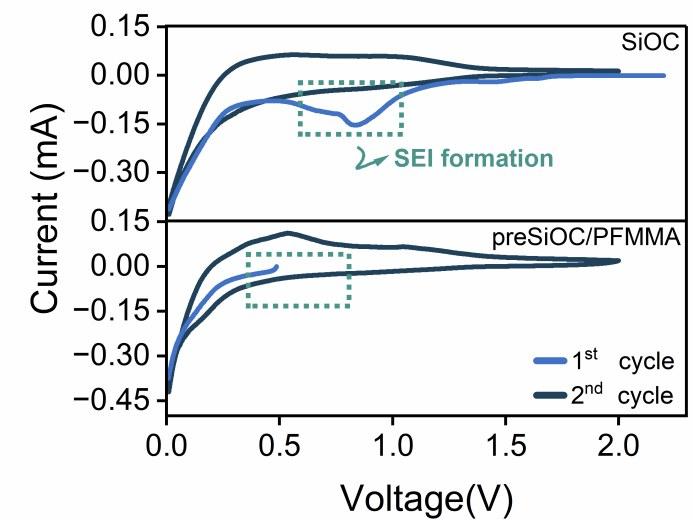


**Figure S21.** Comparative CV profiles of SiOC and preSiOC/PFMMA electrodes.


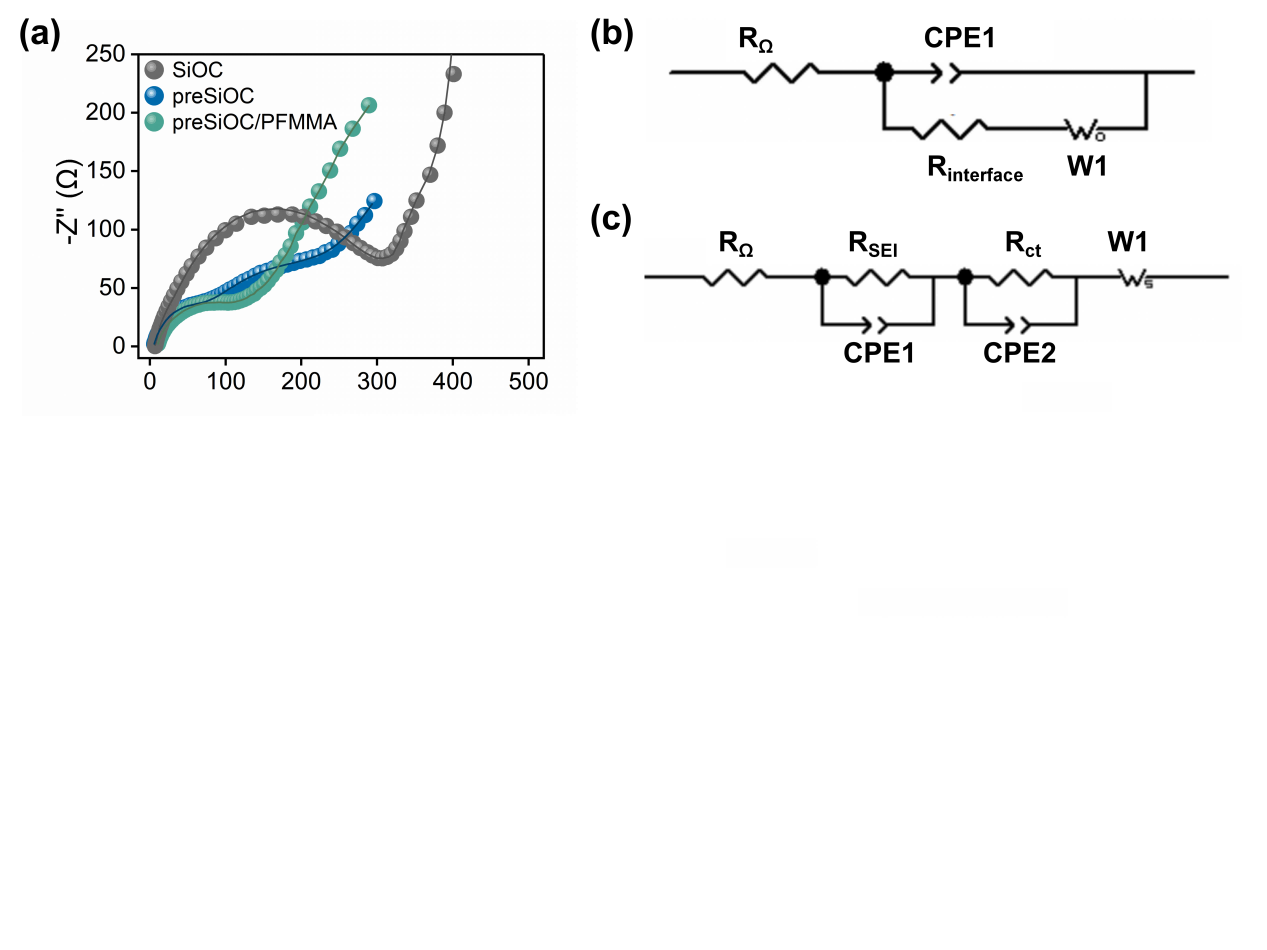


**Figure S22.** (a) EIS spectra of SiOC, preSiOC, and preSiOC/PFMMA electrodes before cycling. (b) Equivalent circuit used for fitting SiOC and preSiOC/PFMMA, for which only one dominant semicircle is observed; the fitted resistance is denoted as *R_Ω_ and* the overall interfacial resistance (*R_interface_*). (c) Equivalent circuit used for fitting preSiOC, which exhibits two distinguishable semicircles and can be resolved into *R_Ω_*, *R_SEI_*, and *R_ct_*. Here, *R_Ω_* is the ohmic resistance, *R_SEI_*, the SEI resistance, *R_ct_*, the charge-transfer resistance, W1 the Warburg diffusion element, and CPE the constant phase element.


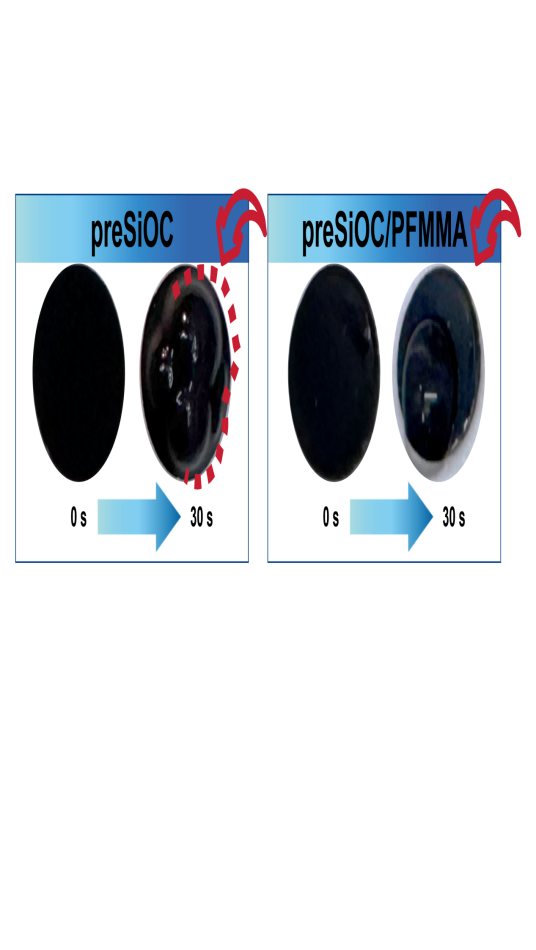


**Figure S23.** Digital photos of preSiOC and preSiOC/PFMMA electrodes, recorded prior to and following contact with water.


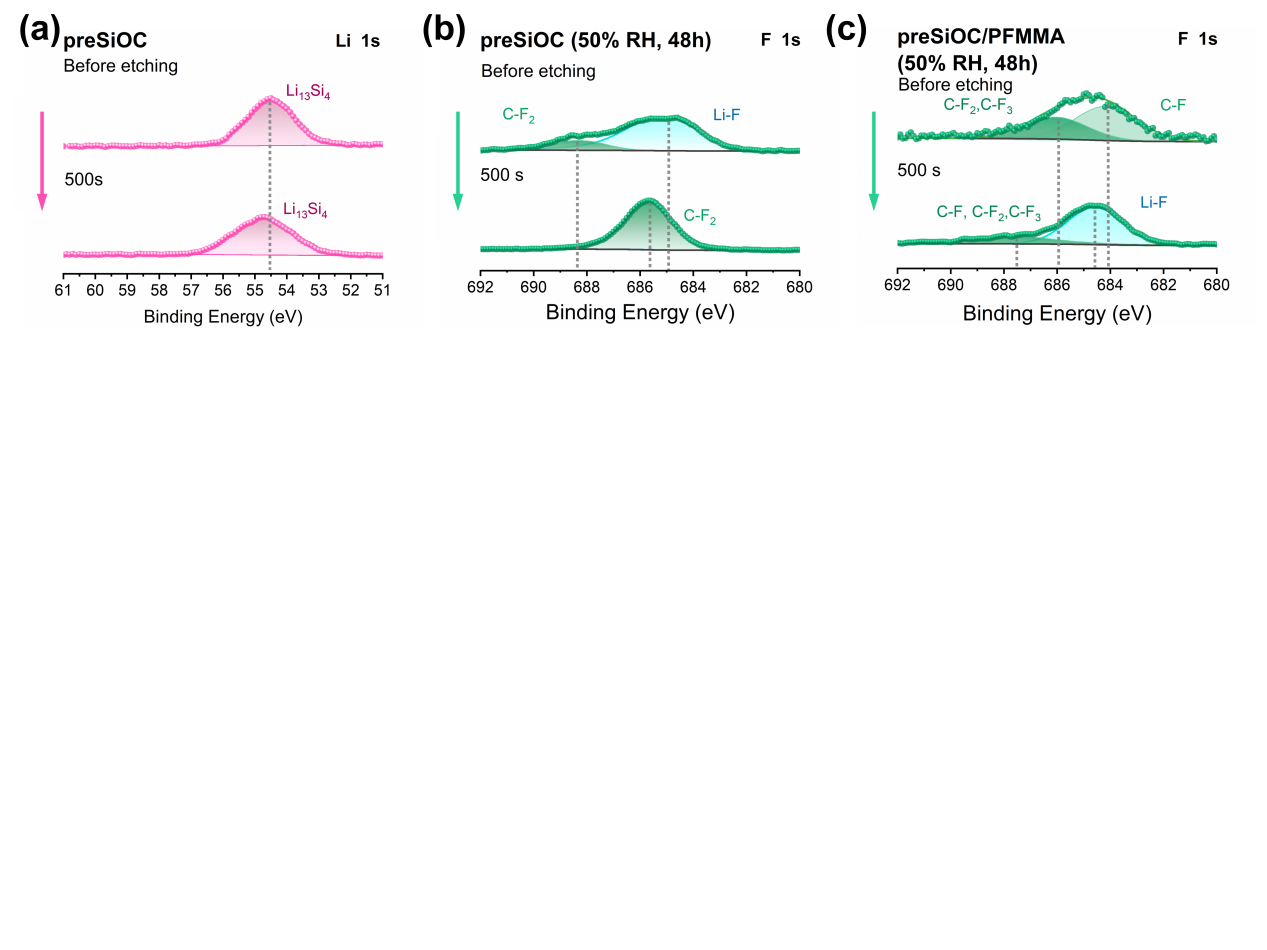


**Figure S24.** (a) XPS spectrum of Li 1s for the pristine preSiOC electrode; (b,c) XPS spectra of F 1s for electrodes after 48 hours ambient exposure: (b) preSiOC, (c) preSiOC/PFMMA.


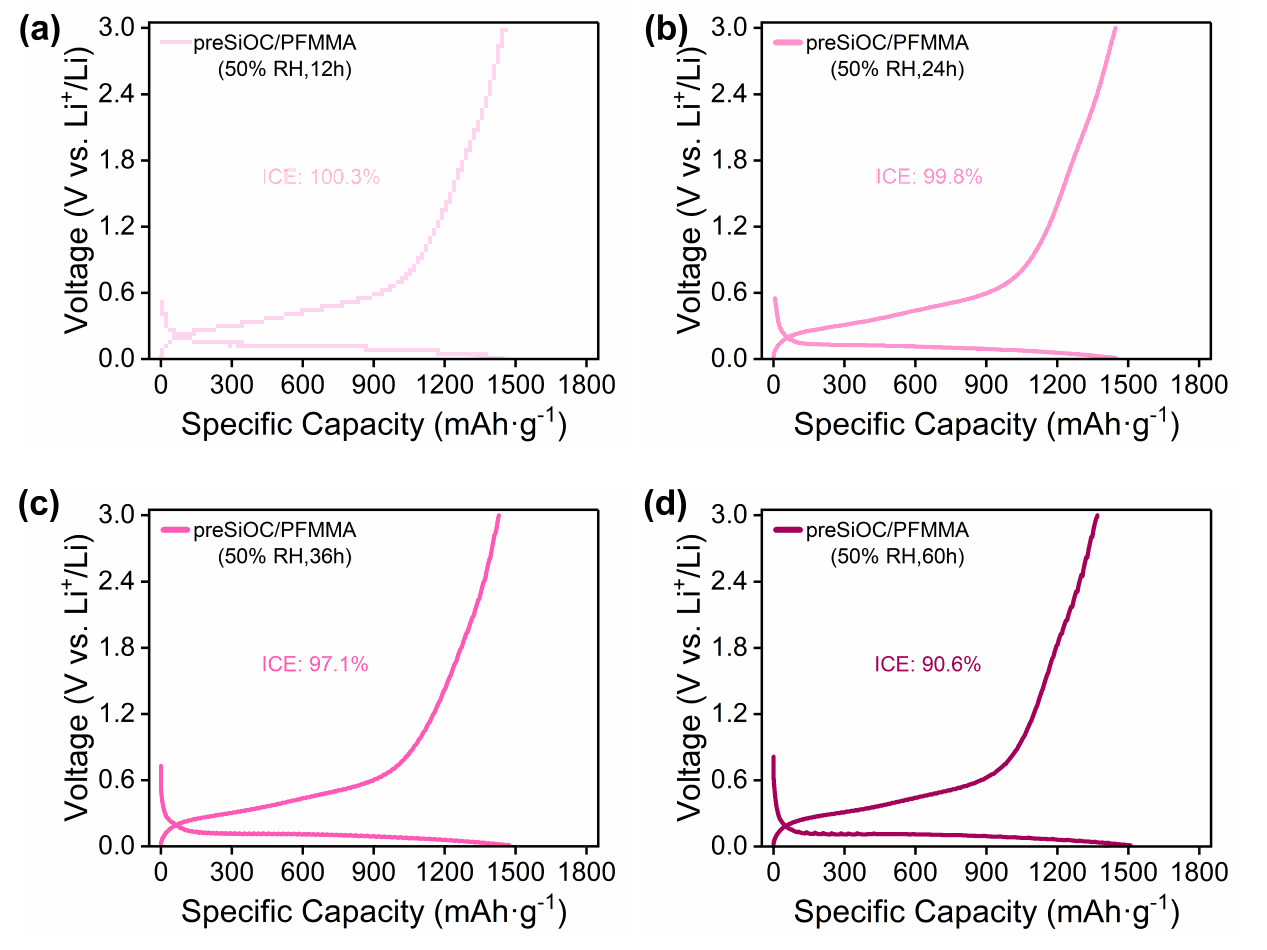


**Figure S25.** The initial charge/discharge profiles preSiOC/PFMMA exposed in ambient air (~50% RH) for (a) 12h, (b) 24h, (c) 36h, (d) 60h.


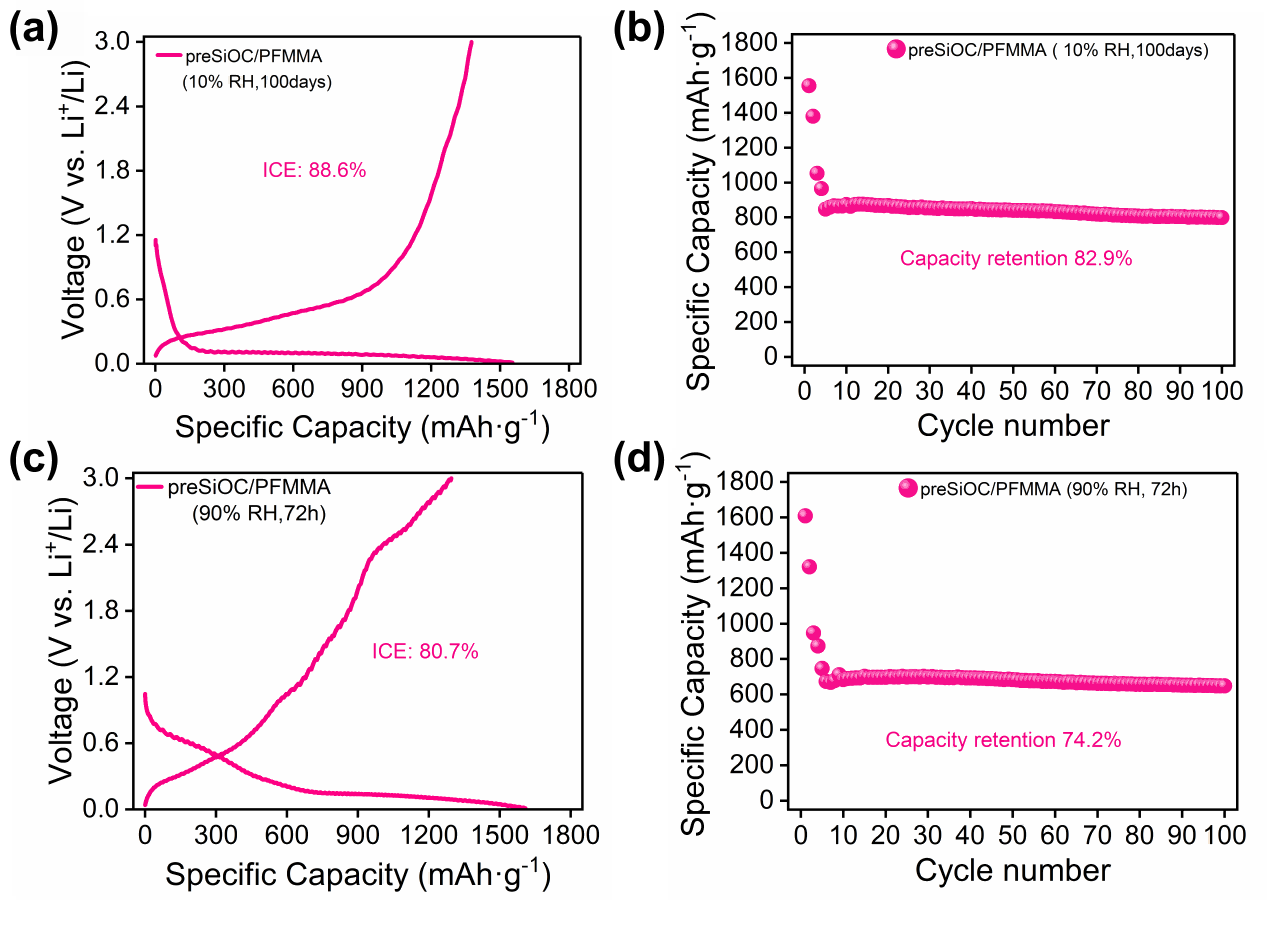


**Figure S26.** The initial charge/discharge profile and cycling performance of preSiOC/PFMMA after exposing to ambient air: (a,b) ~10% RH for 100 days, (c,d) ~90% RH for 3 days.

**
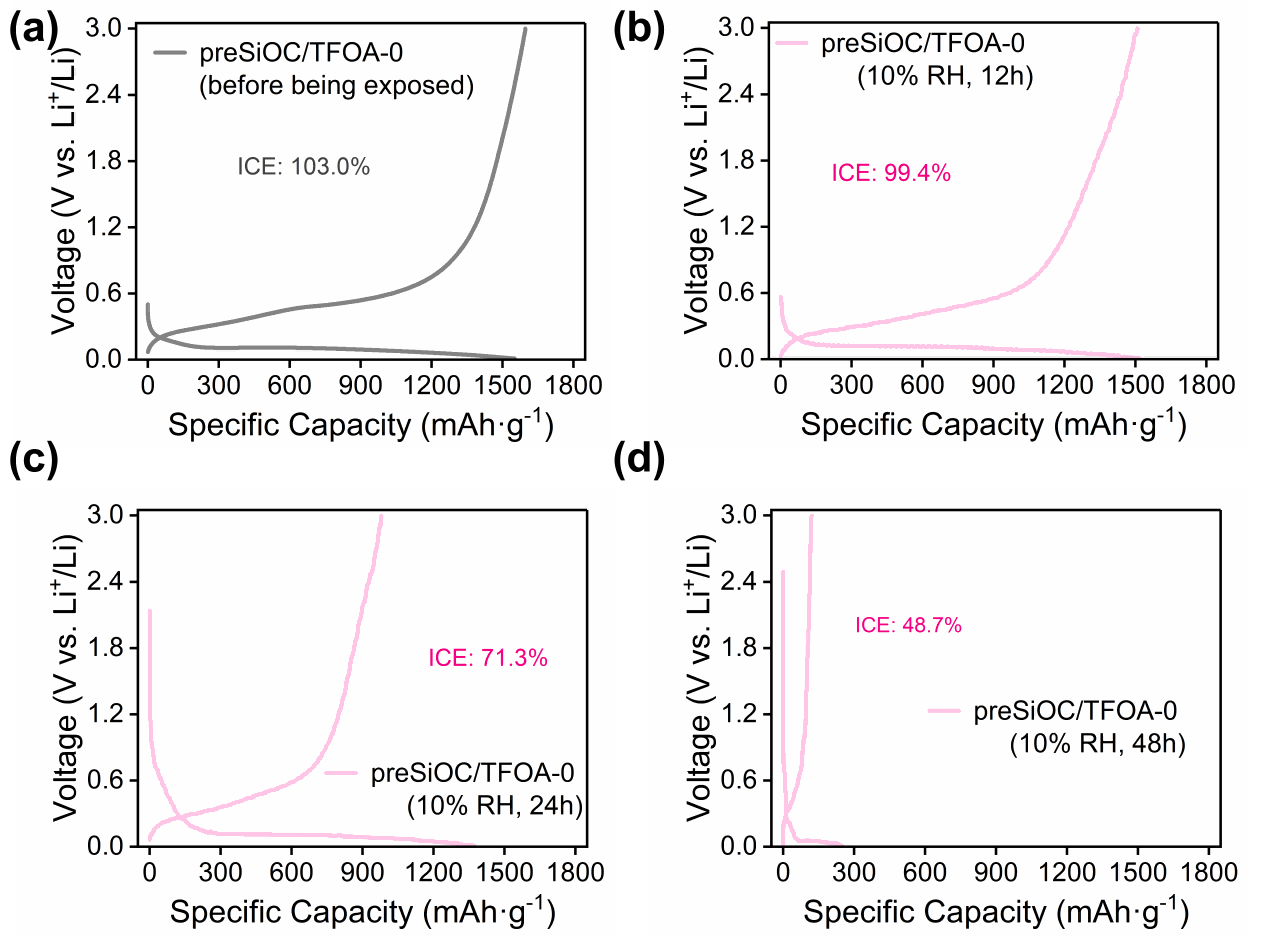
**

**Figure S27.** The first charge/discharge profiles of preSiOC/TFOA-0 before and after exposed to ambient air (~10% RH) for different durations.


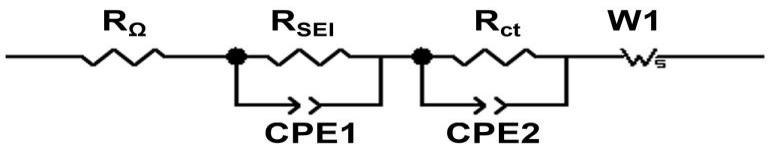


**Figure S28.** Equivalent circuit model used for fitting the EIS data corresponding to Figure 4a.

* *R_Ω_*, ohmic resistance; *R_SEI_*, SEI resistance; *R_ct_*, charge-transfer resistance; CPE_1_ and CPE_2_, constant phase elements corresponding to the SEI/interphase and charge-transfer processes, respectively. Fitted resistance values are now used mainly for comparative analysis of interfacial evolution among different electrodes and cycling states, rather than for over-quantitative interpretation of diffusion-related processes.


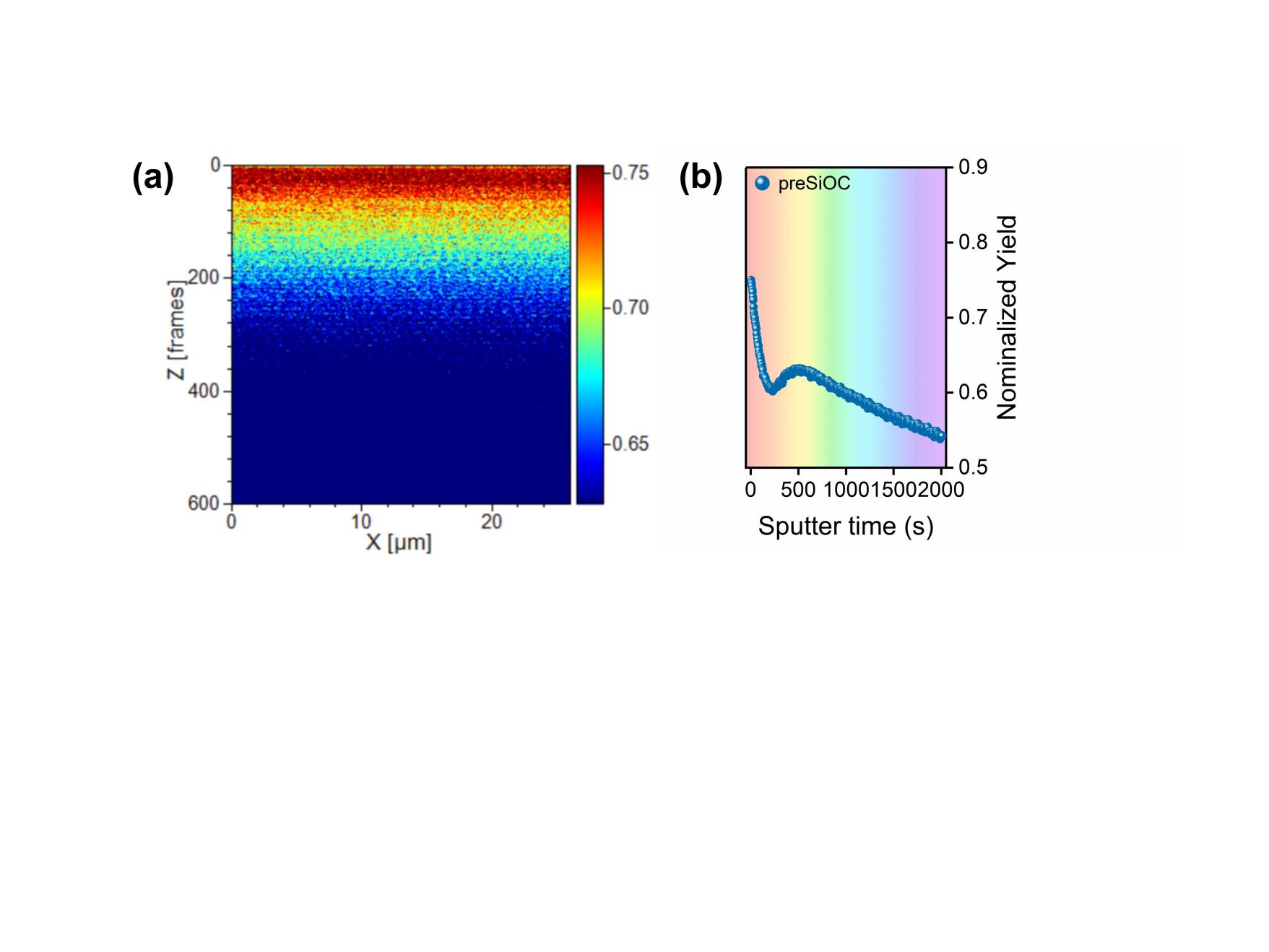


**Figure S29.** TOF-SIMS analysis of preSiOC electrode: (a) spatial distribution maps of F^-^ ions and (b) normalized depth profiles of selected ion fragments.


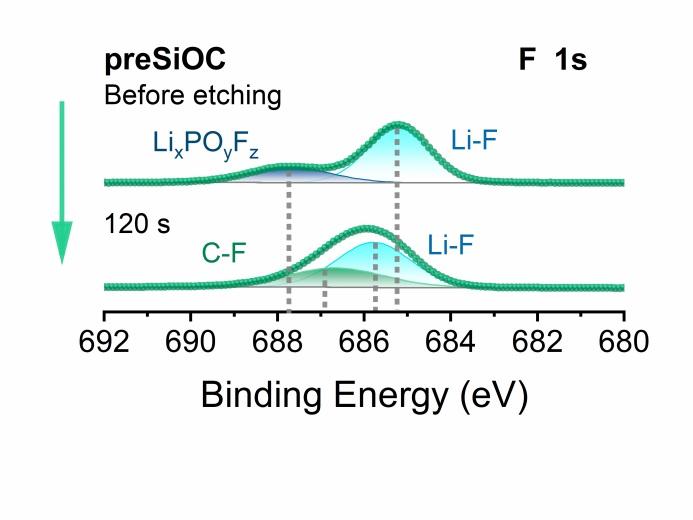


**Figure S30.** XPS spectrum of F 1 s for preSiOC electrode after cycling.

**
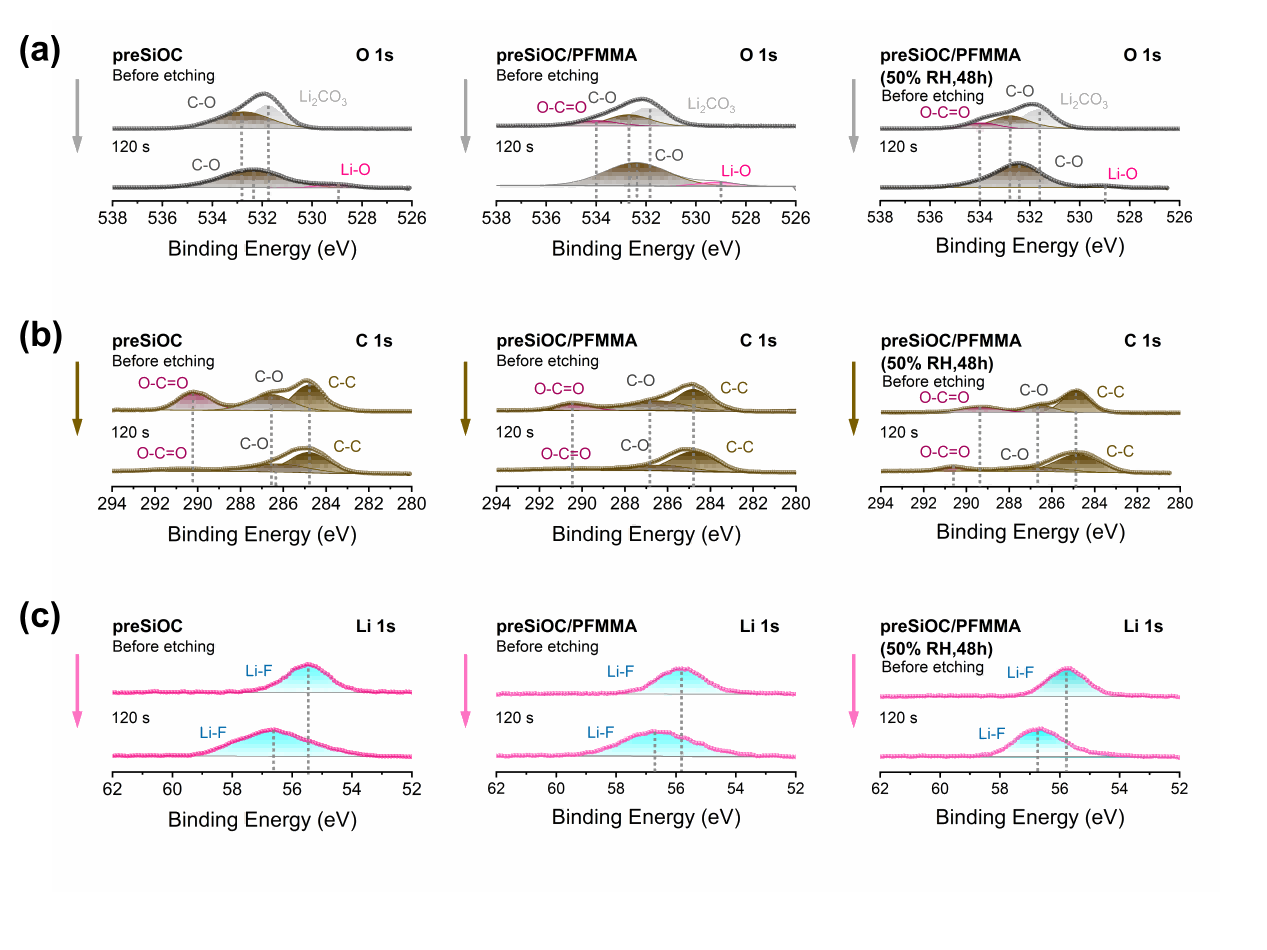
**

**Figure S31.** XPS spectrum of (a) O 1s, (b) C 1s, (c) Li 1s for electrodes after cycling.

**
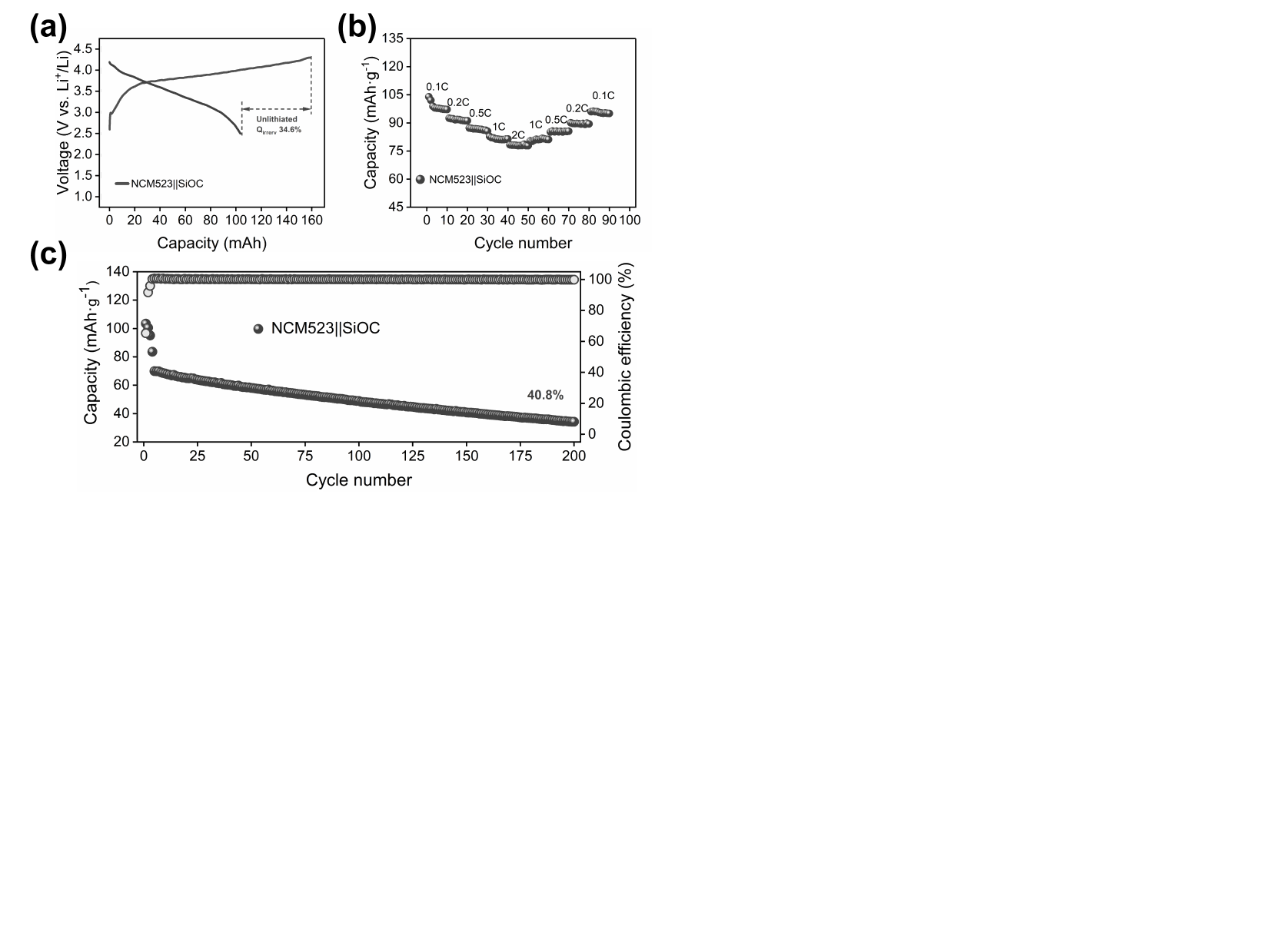
**

**Figure S32.** (a) The initial coulombic efficiency, (b) rate capability at various current densities, and (c) Long-term cycling stability and coulombic efficiency over prolonged operation of NCM523||SiOC.**Table S1.** Surface tension components of water and diiodomethane.

| Liquid | Polar component ${\text{γ}_{\text{L}}}^{\text{P}}\text{(}\text{mJ}\text{/}\text{m}^{\text{2}}\text{)}$ | Dispersive component  ${\text{γ}_{\text{L}}}^{\text{D}}\text{(}\text{mJ}\text{/}\text{m}^{\text{2}}\text{)}$ | Nature |
| --- | --- | --- | --- |
| Water | 51 | 21.8 | Polar |
| Diiodomethane | 2.3 | 48.5 | Nonpolar |

**Table S2.** Contact angle measurements with different probe liquids and the calculated surface free energy of TFOA-0 and PFMMA films.

| Samples | Contact Angle (°) | | $\text{γ}_{\text{L}}$  $\text{(}\text{mJ}\text{/}\text{m}^{\text{2}}\text{)}$ | ${\text{γ}_{\text{S}}}^{\text{D}}$  $\text{(}\text{mJ}\text{/}\text{m}^{\text{2}}\text{)}$ | ${\text{γ}_{\text{S}}}^{\text{P}}$  $\text{(}\text{mJ}\text{/}\text{m}^{\text{2}}\text{)}$ |
| --- | --- | --- | --- | --- | --- |
|  | Water | Diiodomethane |  |  |  |
| TFOA-0 | 89.3 | 43.6 | 72.8 | 37.21 | 0.84 |
| PFMMA | 139.2 | 75.3 | 50.8 | 25.14 | 4.16 |

**Table S3.** Spray-coating parameters for different PFMMA loadings on 20preSiOC electrode.

| **PFMMA Loading (mg)** | **PFMMA Concentration**  **(mg·mL^-1^)** | **Solution Volume**  **(μL)** | **Electrode areal loading (mg·cm^-2^)** |
| --- | --- | --- | --- |
| 0.05 | 4.534 | 500 | 0.0347 |
| 0.1 | 4.134 | 500 | 0.0694 |
| 0.2 | 4.342 | 500 | 0.1389 |
| 0.5 | 4.62 | 500 | 0.3472 |
| 1.0 | 5.279 | 500 | 0.6944 |

**Table S4.** *Equivalent-circuit fitting parameters of* SiOC, preSiOC and preSiOC/PFMMA electrodes derived from the EIS data.

| **Samples** | ***R_Ω_*** | ***R***_interface_ | ***R_ct_*** | ***R_SEI_*** |
| --- | --- | --- | --- | --- |
| **SiOC** | 9.697 | 292.70 | — | — |
| **PreSiOC** | 8.898 | — | 60.86 | 164.72 |
| **PreSiOC/PFMMA** | 8.556 | 71.03 | — | — |

******* For SiOC and preSiOC/PFMMA, which exhibit only one depressed semicircle, the fitted resistance is conservatively denoted as the overall interfacial resistance, *R_interface_*, reflecting the unresolved contribution of ***R_SEI_*** and ***R_ct_*** related processes. In contrast, the two distinguishable semicircles of preSiOC allow separate fitting of ***R_SEI_*** and ***R_ct_***.

**Table S5.** Performance comparison with representative state-of-the-art stabilization approaches for prelithiated electrodes.

| **Prelithiation reagent** | **Capacity (mAh**·**g^-1^)** | **Capacity at different relative humidity**  **(mAh·g^-1^)** | **Duration** | **Capacity retention (%)** |
| --- | --- | --- | --- | --- |
| preSiOC/PFMMA  (this work) | 1457.2 | 1376.5 (10% RH) | 100 days | 94.5% |
|  | 1457.2 | 1418.7 (50% RH) | 48 h | 97.4% |
|  | 1457.2 | 1293.1 (90% RH) | 72 h | 88.7% |
| Artificial-SEI Li_x_Si[10] | 2074 | 1604 (10% RH) | 6 h | 77.34% |
| Li_x_Sn @Ppy NPs[11] | 779 | 768 (10% RH) | 24 h | 98.5% |
| NCM-PMMA-Li_2_O_2_[12] | 1148 | 923 (40% RH) | 8 h | 80.4% |
| ANSBM[13]  (prelithiation reagent) | 688 | 625 (10% RH) | 72h | 90.84% |
|  | 688 | 546 (10% RH) | 720 h | 79.36% |
| ASP-Hp-SiO_x_@C  (prelithiated hollow  sphere)[14] | 1179 | 1058.9 (10~20% RH) | 48 h | 89.8% |
| Surface-passivated Li_3_N (prelithiation reagent)[15] | 1799 | 1612 (12% RH) | 26 h | 89.6% |
| Li_22_Si_5_@C/PVDF-HFP [16] | 1207 | 1200 (30% RH) | 12 h | 99% |
|  | 1207 | 824 (60% RH) | 6 h | 68.2% |
| Li-IBL[17] | 980 | ~892 (85% RH) | 6 h | ~91% |
| HEA@CNT/Li_22_Si_5_@EVA[18] | ~0.32  mAh·cm^-2^ | 0.27 mAh·cm^-2^ (50% RH) | 6 h | 84.37% |
| CLC(sandwiching  LiAg foil between  Two Cu foils)[19] | ~3.0  mAh·cm^-2^ | ~3.0 mAh·cm^-2^ (60% RH) | 12 h | ~100% |

**Table S6.** *R_Ω_* of preSiOC/PFMMA and preSiOC/PFMMA (~50% RH, 48h) electrodes obtained from equivalent circuit fitting of EIS data.

| **Cycle number** | **PreSiOC/PFMMA** | **PreSiOC/PFMMA**  **(50% RH)** |
| --- | --- | --- |
| 10 | 7.299 | 6.911 |
| 20 | 7.132 | 8.592 |
| 30 | 7.055 | 8.158 |
| 40 | 7.558 | 7.650 |
| 50 | 7.099 | 7.787 |
| 60 | 6.798 | 7.761 |
| 70 | 6.665 | 7.628 |
| 80 | 7.194 | 6.823 |
| 90 | 7.121 | 6.857 |
| 100 | 7.586 | 6.827 |

**Table S7.** *R_SEI_* of preSiOC/PFMMA and preSiOC/PFMMA (~50% RH, 48h) electrodes obtained from equivalent circuit fitting of EIS data.

| **Cycle number** | **PreSiOC/PFMMA** | **PreSiOC/PFMMA**  **(50% RH)** |
| --- | --- | --- |
| 10 | 75.12 | 85.99 |
| 20 | 75.03 | 77.60 |
| 30 | 71.67 | 71.55 |
| 40 | 80.25 | 61.17 |
| 50 | 67.99 | 68.55 |
| 60 | 66.36 | 67.27 |
| 70 | 61.90 | 57.37 |
| 80 | 72.43 | 72.06 |
| 90 | 70.45 | 69.49 |
| 100 | 76.34 | 68.28 |

**Table S8.** *R_ct_* of preSiOC/PFMMA and preSiOC/PFMMA (~50% RH, 48h) electrodes obtained from equivalent circuit fitting of EIS data.

| **Cycle number** | **PreSiOC/PFMMA** | **PreSiOC/PFMMA**  **(50% RH)** |
| --- | --- | --- |
| 10 | 568.3 | 717.5 |
| 20 | 552.0 | 630.1 |
| 30 | 609.7 | 666.9 |
| 40 | 733.9 | 818.4 |
| 50 | 739.5 | 897.2 |
| 60 | 852.0 | 967.5 |
| 70 | 869.4 | 982.8 |
| 80 | 843.5 | 978.1 |
| 90 | 817.7 | 949.3 |
| 100 | 865.7 | 920.1 |

**Table S9.** Key parameters relevant to the full cells.

| **Cathode** | | **Anode** | | **PFMMA** |
| --- | --- | --- | --- | --- |
| **Specific Capacity (mAh·g^-1^)** | **Loading**  **(mg·cm^-2^)** | **Specific Capacity (mAh·g^-1^)** | **Loading**  **(mg·cm^-2^)** | **Loading**  **(mg·cm^-2^)** |
| 150 | 9.5 | 1500 | 1.0 | 0.0694 |

**Table S10.** Comparative performance of NCM523||preSiOC, NCM523||preSiOC/PFMMA, and NCM523||preSiOC/PFMMA (50% RH) full-cells prototypes against literature-reported full cells employing Si-based anodes.

| **Anode** | **Cathode** | **Power Density**  **(W·kg^-1^)** | **Energy Density**  **(Wh·kg^-1^)** | **Reference** |
| --- | --- | --- | --- | --- |
| PreSiOC | NCM523 | 257.1 | 417.9 | This work |
| PreSiOC/PFMMA | NCM523 | 255.5 | 388.6 | This work |
| PreSiOC/PFMMA  (50% RH) | NCM523 | 249.45 | 366.3 | This work |
| HEA@CNT/Li_22_Si_5_  @EVA-Cu | NCM811 | 53.5 | 535 | [S18][18] |
| prelithiated-Si@G/C | NCM811 | 233.1 | 466.1 | [S20][20] |
| HC8/N-Si_2_ | NCM523 | 35 | 348 | [S21][21] |

****Energy and power densities are calculated on the* active materials.**

**Reference:**

[1] G. Kresse, J. Furthmüller, *Phys. Rev. B* **1996**, *54*, 11169.

[2] G. Kresse, D. Joubert, *Phys. Rev. B* **1999**, *59*, 1758.

[3] U. Essmann, L. Perera, M.L. Berkowitz, T. Darden, H. Lee, L.G. Pedersen, *J. Chem. Phys.* **1995**, *103*, 8577.

[4] W.F. Van Gunsteren, H.J.C. Berendsen, *Mol. Simul.* **1988**, *1*, 173.

[5] G. Bussi, D. Donadio, M. Parrinello, *J. Chem. Phys.* **2007**, *126*, 014101.

[6] H.J.C. Berendsen, J.P.M. Postma, W.F. van Gunsteren, A. DiNola, J.R. Haak, *J. Chem. Phys.* **1984**, *81*, 3684.

[7] S. Shalel-Levanon, A. Marmur, *J. Colloid Interface Sci.* **2003**, *262*, 489.

[8] A. Burdzik, M. Stähler, M. Carmo, D. Stolten, *Int. J. Adhes.* **2018**, *82*, 1.

[9] D. Su, X. Wang, H.-W. Yang, C. Hong, *Powder Technol.* **2019**, *356*, 423.

[10] J. Zhao, Z. Lu, H. Wang, W. Liu, H.-W. Lee, K. Yan, D. Zhuo, D. Lin, N. Liu, Y. Cui, *J. Am. Chem. Soc.* **2015**, *137*, 8372.

[11] S. Li, C. Wang, J. Yu, Y. Han, Z. Lu, *Energy Storage Mater.* **2019**, *20*, 7.

[12] L. Zheng, A. Yu, G. Li, J. Zhang, *ACS Appl. Mater. Interfaces* **2022**, *14*, 38706.

[13] L. Liu, X. Zuo, Y. Cheng, Y. Xia, *ACS Appl. Mater. Interfaces* **2022**, *14*, 28748.

[14] F. Wang, B. Wang, Z. Yu, C. Zhu, P. Liu, J. Li, B. Wang, Y. Zhou, D. Wang, H.K. Liu, S. Dou, Cell Rep. Phys. Sci., **2022**, *3*, 100872.

[15] C. Liu, H. Zhang, T. Li, W. Liu, C. Qu, X. Yang, X. Li, *J. Energy Storage* **2024**, *99*, 113256.

[16] H. Wang, A. Shao, R. Pan, W. Tian, Q. Jia, M. Zhang, M. Bai, Z. Wang, F. Liu, T. Liu, X. Tang, S. Li, Y. Ma, *ACS Nano* **2023**, *17*, 21850.

[17] H. Wang, M. Zhang, Q. Jia, D. Du, F. Liu, M. Bai, W. Zhao, Z. Wang, T. Liu, X. Tang, S. Li, Y. Ma, *Nano Energy* **2022**, *95*, 107026.

[18] H. Wang, Y. Yuan, Q. Jia, A. Shao, M. Zhang, Z. Wang, L. Cheng, X. Tang, S. Li, Y. Ma, *Adv. Funct. Mater.* **2024**, *34*, 2314186.

[19] C. Zhang, X. Chen, W. Wan, G. Liu, Q. Nie, F. Yang, X. Li, S. Li, Y. Huang, C. Wang, *Energy Environ. Sci.* **2024**, *17*, 6766.

[20] M. Bai, L. Yang, Q. Jia, X. Tang, Y. Liu, H. Wang, M. Zhang, R. Guo, Y. Ma, *ACS Appl. Mater. Interfaces* **2020**, *12*, 47490.

[21] Y. Abe, I. Saito, M. Tomioka, M. Kabir, S. Kumagai, *Batteries* **2022**, *8*, 210.
